# Group 2 Innate Lymphoid Cells Exhibit a Dynamic Phenotype in Allergic Airway Inflammation

**DOI:** 10.3389/fimmu.2017.01684

**Published:** 2017-12-01

**Authors:** Bobby W. S. Li, Ralph Stadhouders, Marjolein J. W. de Bruijn, Melanie Lukkes, Dior M. J. M. Beerens, Maarten D. Brem, Alex KleinJan, Ingrid Bergen, Heleen Vroman, Mirjam Kool, Wilfred F. J. van IJcken, Tata Nageswara Rao, Hans Jörg Fehling, Rudi W. Hendriks

**Affiliations:** ^1^Department of Pulmonary Medicine, Rotterdam, Netherlands; ^2^Center for Biomics, Erasmus MC Rotterdam, Rotterdam, Netherlands; ^3^Institute of Immunology, University Clinics Ulm, Ulm, Germany

**Keywords:** allergy, asthma, IL-33, group 2 innate lymphoid cell, house dust mite, mouse model

## Abstract

Group 2 innate lymphoid cells (ILC2) are implicated in allergic asthma as an early innate source of the type 2 cytokines IL-5 and IL-13. However, their induction in house dust mite (HDM)-mediated airway inflammation additionally requires T cell activation. It is currently unknown whether phenotypic differences exist between ILC2s that are activated in a T cell-dependent or T cell-independent fashion. Here, we compared ILC2s in IL-33- and HDM-driven airway inflammation. Using flow cytometry, we found that surface expression levels of various markers frequently used to identify ILC2s were dependent on their mode of activation, highly variable over time, and differed between tissue compartments, including bronchoalveolar lavage (BAL) fluid, lung, draining lymph nodes, and spleen. Whereas *in vivo* IL-33-activated BAL fluid ILC2s exhibited an almost uniform CD25^+^CD127^+^T1/ST2^+^ICOS^+^KLRG1^+^ phenotype, at a comparable time point after HDM exposure BAL fluid ILC2s had a very heterogeneous surface marker phenotype. A major fraction of HDM-activated ILC2s were CD25^low^CD127^+^T1/ST2^low^ ICOS^low^KLRG1^low^, but nevertheless had the capacity to produce large amounts of type 2 cytokines. HDM-activated CD25^low^ ILC2s in BAL fluid and lung rapidly reverted to CD25^high^ ILC2s upon *in vivo* stimulation with IL-33. Genome-wide transcriptional profiling of BAL ILC2s revealed ~1,600 differentially expressed genes: HDM-stimulated ILC2s specifically expressed genes involved in the regulation of adaptive immunity through B and T cell interactions, whereas IL-33-stimulated ILC2s expressed high levels of proliferation-related and cytokine genes. In both airway inflammation models ILC2s were present in the lung submucosa close to epithelial cells, as identified by confocal microscopy. In chronic HDM-driven airway inflammation ILC2s were also found inside organized cellular infiltrates near T cells. Collectively, our findings show that ILC2s are phenotypically more heterogeneous than previously thought, whereby their surface marker and gene expression profile are highly dynamic.

## Introduction

The capability of group 2 innate lymphoid cells (ILC2) to secrete large amounts of IL-5 and IL-13 has led to the investigation of their role in the pathogenesis of allergic diseases in recent years (1, 2). ILCs are a family of effector lymphocytes that do not express antigen receptors. They are classified according to their transcription factor requirements and distinct cytokine secreting patterns that mirror the profiles of T helper subsets (3, 4). ILC2s were originally characterized as an IL-25- and IL-33-responsive cell population that provides a critical early source of type 2 cytokines for the expulsion of parasitic worms (5–8). Like other ILC family members, ILC2s develop from the common lymphoid progenitors (CLP) and lack classic hematopoietic lineage markers and are thus defined as Lineage negative (9, 10). They express Thy-1 (CD90), c-Kit (CD117) and Sca-1 as well as a broad range of cytokine receptors, including IL-7Rα (CD127), IL-2Rα (CD25), IL-25R (IL-17RB), and IL-33R (T1/ST2), leading to frequent use of these markers to identify and isolate ILC2s (11–13).

When stimulated by epithelial cell-derived innate cytokines, such as IL-25, IL-33, and thymic stromal lymphopoietin (TSLP), prostaglandin D2 from mast cells or cysteinyl leukotrienes secreted by activated hematopoietic cells, ILC2s rapidly expand and secrete large amounts of type 2 cytokines (1, 2, 14). Accordingly, intranasal administration of IL-25 or IL-33 induces eosinophilic airway inflammation and expansion of ILC2s, independently of B or T cells (15–17). Mouse models of allergic airway inflammation induced by papain and *Alternaria* have shown rapid release of IL-25 and IL-33 followed by robust ILC2 induction prior to T cell activation, suggesting an early sentinel function (16, 18–20). In contrast to these studies, exposure to other allergens such as *Aspergillus* and house dust mite (HDM) indicates a prominent role of T cells in the initiation of allergic inflammation (21, 22). We have previously shown that, in HDM-induced allergic inflammation, ILC2 induction requires T cell activation. Although accumulation of ILC2s in the bronchoalveolar lavage (BAL) fluid is independent of IL-33, cytokine production by ILC2s is markedly reduced in IL-33 knockout mice (22). Additionally, T cell-derived IL-21 promotes type 2 immunity to HDM and blockade of CD28 signaling during HDM exposure represses airway hyperreactivity and lung inflammation (23, 24), further supporting that both IL-33 and T cells are necessary for full ILC2 responses. Evidence for direct interactions between T cells and ILC2s includes the expression of MHC class II and co-stimulatory molecules such as CD86 and ICOS/ICOS-L by ILC2s (25–27). Taken together, these studies indicate the involvement of a complex array of signals and interactions for the activation of ILC2s in allergy. Importantly, ILC2s have mainly been studied in models in which they are strongly and rapidly activated in a T cell-independent fashion, but the phenotypic characteristics of ILC2s induced in T cell-dependent inflammation, including HDM-mediated allergic airway inflammation models, is currently not clear. Studies using IL-5 and IL-13 reporter mice have shown that in unstimulated conditions or upon IL-33 stimulation pulmonary ILC2s are mainly localized in the lung submucosa close to epithelial cells in collagen-rich regions near blood vessels and airways (28, 29). However, ILC2 localization within a more physiological airway inflammation and their localization relative to Th2 cells remain unknown.

Plasticity of ILCs has first been reported in intestinal group 3 innate lymphoid cells (ILC3), which downregulate RORγt expression and simultaneously upregulate T-bet to transform into a group 1 innate lymphoid cell (ILC1)-like phenotype depending on IL-12, IL-18, and IL-7 (30). Conversely, ILC1s can trans-differentiate into ILC3s in the presence of IL-1β and IL-23 (31). ILC2s are also able to upregulate T-bet under influence of IL-33 and IL-1β and can produce IFN-γ, whereby retention of IL-13 producing capabilities resulting in a hybrid ILC1/ILC2 phenotype has been reported (32–35). Heterogeneity and plasticity in relation to environmental signals have recently been substantiated by single-cell transcriptome analyses (36–38). Taken together, these publications demonstrate the importance of micro-environmental cues for the function of ILC2s. As a result, the expression of cytokines and cytokine receptors by ILC2s may depend on their manner of activation and may differ between tissues. Thus, we relied on transcription factor GATA3 as a key ILC2 marker, which is central to ILC2 development and function and is constitutively expressed at high levels (39). We have previously reported dose-dependent effects of GATA3 both on ILC2 development from CLPs and on ILC2 function in allergic airway inflammation (40, 41). GATA3 additionally plays a major role as a master regulator of Th2 cell differentiation and drives the early development of other ILC subsets from the common ILC progenitor (42–44).

Although plasticity of ILC2 is studied in the context of their capacity to trans-differentiate into other types of ILCs, it remains unknown how the ILC2 phenotype is dependent on activation status, how it develops over time, what the differences are between various tissue compartments, and how stable or reversible the ILC2 phenotype is. In this report, we aimed to compare the dynamics and kinetics of the ILC2 phenotype in the context of T cell-independent and T cell-dependent airway inflammation using IL-33 and HDM, respectively. We employed a novel *Gata3*^YFP/YFP^ mouse strain, named GATA3 IRES Reporter (GATIR), which allows for synchronous transcription of GATA3 and yellow fluorescent protein (YFP) as separate proteins without affecting GATA3 protein levels or function (Tata Nageswara Rao and Hans Jörg Fehling, manuscript in preparation). These mice enabled us to characterize and compare the phenotype of ILC2s in detail in acute and chronic mouse models of airway inflammation and to evaluate their localization in the lungs during an inflammatory response relative to other immune cells.

## Materials and Methods

### Mice

Wild-type C57BL/6 mice were purchased from Envigo (United Kingdom). The GATIR mouse strain, in which an IRES-YFP sequence was inserted into the 3′ untranslated region of the *Gata3* gene resulting in concomitant production of GATA3 and YFP protein, was on a C57BL/6 background (Tata Nageswara Rao and Hans Jörg Fehling, manuscript in preparation). Only homozygous GATIR knock-in mice were used for analysis. *Gata3^+^*^/^*^−^* mice (C57BL/6), in which one of the *Gata3* alleles is targeted by a lacZ reporter, have been described previously (45). Mice were ~8–16 weeks old at time of analysis. All animals were housed at the Erasmus MC Animal Center under specific pathogen-free conditions. All experiments were approved by the Erasmus MC Animal Ethics Committee.

### Induction of Airway Inflammation

For IL-33-induced airway inflammation, mice were anesthetized using isoflurane and treated three times intranasally or intratracheally every other day with 0.5 µg IL-33 (BioLegend, USA) or PBS (GIBCO Life Technologies, USA) as described previously (17). Mice were sacrificed at the indicated time points after the final IL-33 administration.

For acute HDM-induced allergic airway inflammation, mice were anesthetized using isoflurane and sensitized by intranasal or intratracheal injection with 1 or 10 µg HDM extract (Greer, SC, USA) or PBS. After 7 days, animals were challenged daily on days 0–4 with 10 µg HDM intranasally or intratracheally and sacrificed at the indicated time points after the final treatment as described previously (46). For chronic HDM-induced allergic airway inflammation, mice were anesthetized using isoflurane and treated intranasally three times weekly for 5 weeks with 25 µg HDM or PBS and sacrificed at the indicated time points after the final treatment [adapted from Ref. (47)].

### Flow Cytometry

Single cell suspensions were prepared from lungs, lymph nodes, and spleens by mechanical disruption of the tissues without digestive enzymes using a 100-µm cell strainer (BD Biosciences, USA) in PBS containing 0.5% bovine serum albumin and 5 mM EDTA (Sigma-Aldrich, USA). Bone marrow cells were extracted from the femur and BAL fluid was obtained by flushing the lungs three times with 1 mL PBS containing 0.5 mM EDTA. Intracellular cytokine production was measured upon stimulation of cells at 37°C with phorbol 12-myristate 13-acetate (PMA) and ionomycin (Sigma-Aldrich, USA), supplemented with GolgiStop (BD Biosciences, USA) for 4 h prior to staining. Flow cytometry surface and intracellular staining procedures have been described previously (17, 41). A comprehensive list of antibodies used for flow cytometry is presented in Table S1 in Supplementary Material. Lineage-negative cells were defined as cells not expressing CD3, CD4, CD5, CD8α, CD11b, CD11c, CD19, B220, NK1.1, FcεRIα, TER-119, and Gr-1. Data were acquired using a LSR II flow cytometer equipped with three lasers and FACSDiva (BD Biosciences, USA) and analyzed by FlowJo (Tree Star, Inc., USA).

### ILC2 Cell Sorting and RNA-Seq

For FACS sorting, BAL fluid cells were stained with fluorescently labeled monoclonal antibodies. ILC2 fractions were sorted as Lineage^−^Sca-1^+^YFP^+^ cells using a FACSAria flow cytometer equipped with three lasers and FACSDiva software (Beckton Dickinson). Data analysis was performed with FlowJo software (Tree Star, Inc.).

RNA was extracted using the RNeasy Micro kit (Qiagen) according to the manufacturer’s instructions. Library preparation was performed using the Smart-seq2 methodology (48) and sequenced according to the Illumina TruSeq Rapid v2 protocol on an Illumina HiSeq2500 (single read mode, 51 bp read length). Reads were aligned to the mouse genome (mm10 build) using HISAT2 (49). Sample scaling and statistical analysis were performed using the R package DESeq2 (50) as implemented in HOMER (51); genes with >1 absolute log2-fold change and adjusted *p* < 0.01 (Wald test) were considered differentially expressed. Standard Reads Per Kilobase Million (RPKM) values were used as an absolute measure of gene expression. Genes with average RPKM <2 were considered not expressed. Pathway analyses on differentially expressed genes were performed using Metascape^1^ (52).

### Confocal Microscopic Imaging

Lungs from GATIR mice were inflated with OCT embedding medium containing 2% PFA (Thermo Fisher Scientific, USA) and snap frozen in liquid nitrogen to preserve morphology and YFP fluorescence. Lymph nodes were fixed in 2% PFA, placed in 30% sucrose in PBS overnight and embedded in OCT and stored at −80°C. 7-µm thick cryosections were cut at −20°C using a cryostat (Thermo Fisher Scientific) and stained at room temperature with primary antibodies for 1 h and secondary antibodies for 30 min. A comprehensive list of antibodies used for confocal microscopy is presented in Table S2 in Supplementary Material. Slides were stained with DAPI (Invitrogen, USA) for 5 min and sealed with Vectashield (Vector Laboratories, USA) and examined with an LSM 510 Meta confocal microscope equipped with a 405, 488, 543, and 633 nm laser (Zeiss, Germany). Images were processed and analyzed in Fiji, an open source scientific image processing application based on ImageJ.^2^

### Statistical Analysis

Statistical comparisons were performed by Mann–Whitney *U* tests and a *p*-value of <0.05 was considered statistically significant. All analyses were performed using Prism (GraphPad Software, USA).

## Results

### The GATIR Knock-In Does Not Affect GATA3 Expression Levels

We aimed to make use of expression of the *Gata3* gene as a central ILC2 marker that is independent of cell surface proteins on ILC2s. We have previously shown that GATA3 is critical for ILC2 development in a dose-dependent manner (40). Reduction of GATA3 levels as in *Gata3^+/−^* mice did not detectably affect splenic or thymic CD4^+^ and CD8^+^ T cells in steady state (Figure S1A in Supplementary Material). However, a negative impact was observed on IL-33-induced ILC2 accumulation and cytokine production and the associated eosinophilia in the BAL fluid of mice (Figure S1B in Supplementary Material). Similar effects were found in an HDM-induced mouse model for airway inflammation, whereby reduced GATA3 levels also affected CD4^+^ T cell accumulation and their cytokine content in the BAL fluid (Figure S1C in Supplementary Material). Despite normal development of CD4^+^ and CD8^+^ T cells and normal *in vitro* proliferation of naïve CD4^+^ T cells following αCD3/αCD28-mediated stimulation, *Gata3^+/−^* T cells showed a significant decline in type 2 cytokine production *in vitro*, indicating impaired Th2 differentiation (Figures S1D,E in Supplementary Material).

**Figure 1:**
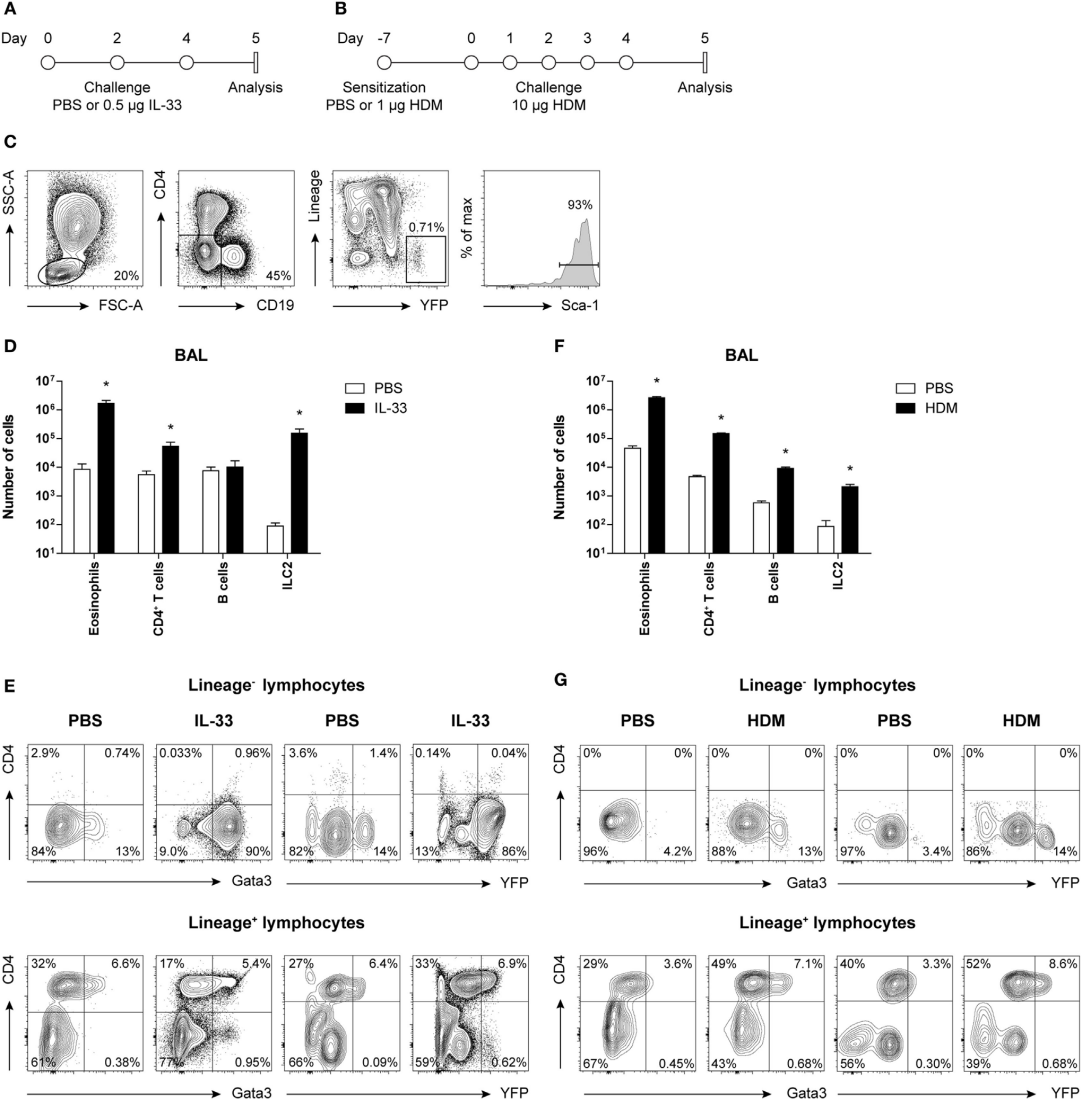
Concomitant GATA3 and yellow fluorescent protein (YFP) expression in group 2 innate lymphoid cells (ILC2s) after IL-33 and house dust mite (HDM) stimulation. **(A,B)** Schemes for intranasal **(A)** IL-33 and **(B)** HDM treatment of GATA3 IRES Reporter mice for induction of acute airway inflammation. Mice were treated with PBS, IL-33, or HDM at indicated time points and analysis was performed 1 day after the final challenge. **(C)** Flow cytometric identification of Lineage^−^YFP^+^Sca-1^+^ ILC2s in bronchoalveolar lavage (BAL) fluid. **(D,F)** Quantification of eosinophils, CD4^+^ T cells, B cells, and ILC2s in the BAL fluid of IL-33 and HDM-treated mice. Data are shown as mean + SEM of *n* = 3–5 mice per group of a single experiment and are representative of two independent experiments. *p ≤ 0.05 compared to PBS control unless otherwise indicated. **(E,G)** Analysis of intracellular GATA3 and YFP expression by flow cytometry in Lineage^−^ and Lineage^+^ lymphocytes from BAL fluid of IL-33 and HDM-treated mice. Plots represent combined data using the concatenate option in FlowJo (*n* = 3–5), representative of two independent experiments.

It was, therefore, critical that the *Gata3* reporter mice to be used for visualization and phenotypic characterization of ILC2s and Th2 cells had unaltered GATA3 expression. GATIR mice harbor an IRES-YFP sequence that was inserted into the 3′ untranslated region of the *Gata3* gene, allowing for the production of YFP as a separate protein concomitant with *Gata3* transcription (Tata Nageswara Rao and Hans Jörg Fehling, manuscript in preparation). We administered either IL-33 or HDM intranasally to GATIR mice to induce ILC2 activation and airway inflammation in a T cell-independent and T cell-dependent manner, respectively (Figures 1A,B).

Group 2 innate lymphoid cells were defined by flow cytometry as Lineage^−^ lymphocytes expressing YFP and Sca-1 (Figure 1C). Analysis of BAL fluid obtained 1 day after the final challenge showed that IL-33 triggered expansion of eosinophils, ILC2s, and to a lesser extent CD4^+^ T cells (Figure 1D). The YFP signals paralleled the expression of GATA3 protein, as detected by intracellular flow cytometry using GATA3-specific antibodies, both within Lineage^−^ and Lineage^+^ lymphocyte fractions (Figure 1E).

In HDM-mediated allergic airway inflammation B cells were also expanded, in addition to eosinophils, CD4^+^ T cells, and ILC2s (Figure 1F), and a similar correlation between GATA3 expression and YFP signals was found in both Lineage^−^ and Lineage^+^ lymphocyte fractions (Figure 1G). Furthermore, the influx of eosinophils and ILC2s in BAL fluid after IL-33 administration did not differ between GATIR mice and wild-type controls. Upon IL-33 exposure, the mean fluorescence intensity (MFI) values of intracellular GATA3 in ILC2s were comparable between the two groups of mice in BAL fluid, lung, and mediastinal lymph node (MLN) (Figure S2A in Supplementary Material). Likewise, GATIR mice paralleled wild-type counterparts when exposed to HDM and no differences in the induction of eosinophils and CD4^+^ T cells and GATA3 expression within Th2 cells were observed (Figure S2B in Supplementary Material). Taken together, these data demonstrate that YFP provides an accurate reflection of intracellular GATA3 expression, although a direct comparison between intracellular GATA3 protein and YFP levels could not be accomplished due to the loss of YFP signal during cell fixation required for GATA3 detection. We conclude that GATA3 protein levels and ILC2 and Th2 induction are not affected by the inserted IRES-YFP sequence, enabling us to use YFP expression in GATIR mice for the identification of ILC2s.

### IL-33 and HDM-Stimulated ILC2s Display a Variable, Unstable, and Heterogeneous Surface Phenotype

**Figure 2:**
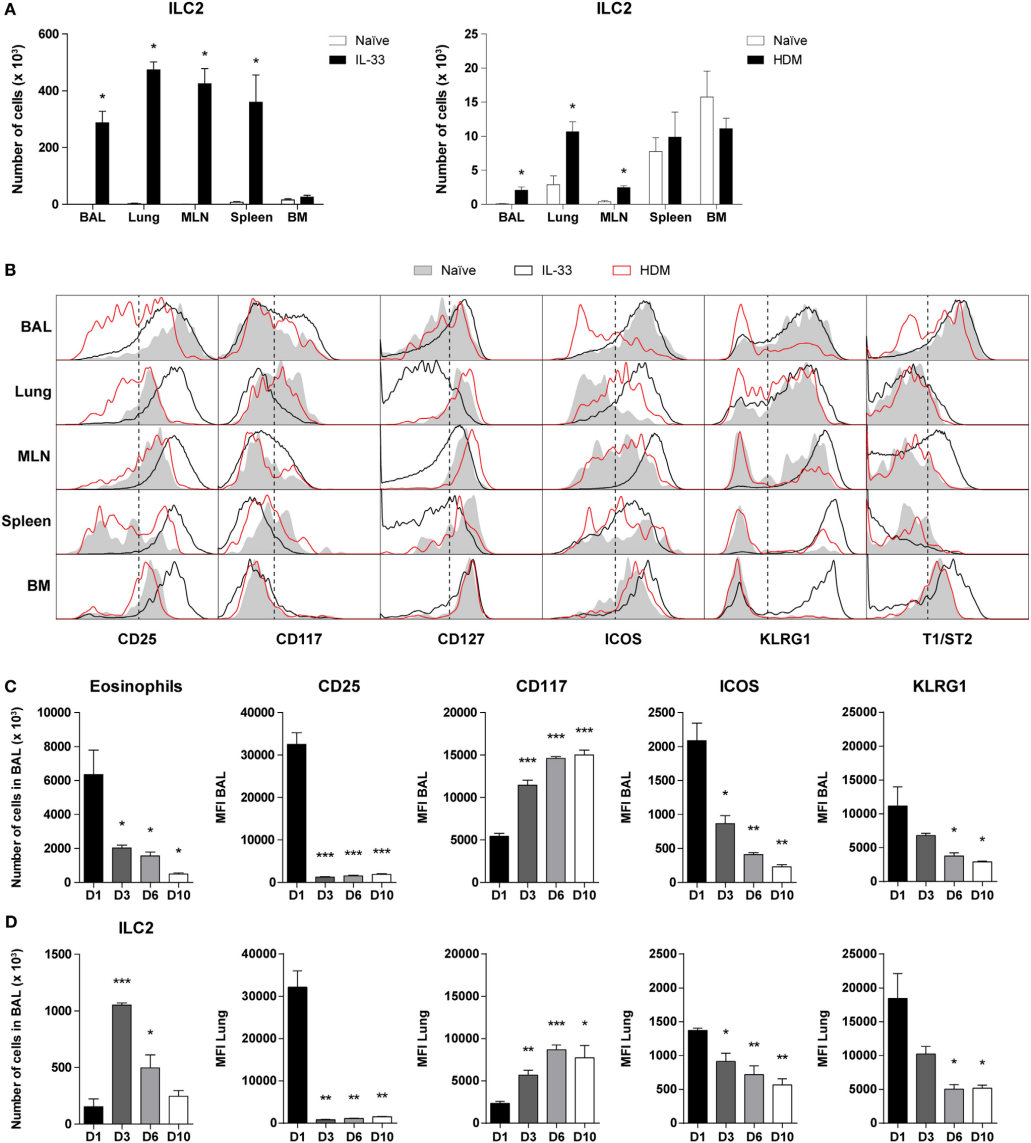
Dynamics of phenotypic group 2 innate lymphoid cell (ILC2) heterogeneity in airway inflammation. **(A)** Quantification of ILC2s in bronchoalveolar lavage (BAL) fluid, lung, mediastinal lymph node (MLN), spleen, and bone marrow (BM) of GATA3 IRES Reporter mice treated with IL-33 and house dust mite (HDM). **(B)** Comparison of ILC2 phenotype in the indicated tissues derived from naïve (*shaded*), IL-33 (*black*), or HDM-treated (*red*) mice. Plots represent combined data using the concatenate option in FlowJo (*n* = 4–6), representative of two independent experiments. **(C)** Eosinophil and ILC numbers in BAL fluid of IL-33-treated mice at days 1, 3, 6, and 10 after the final challenge. **(D)** Quantification of BAL fluid and lung ILC2 surface markers expressed as mean fluorescence intensity (MFI) values. **(A,C,D)** Data are shown as mean + SEM of *n* = 4–6 mice per group of a single experiment and are representative of two independent experiments. *p ≤ 0.05, **p ≤ 0.01, ***p ≤ 0.001 compared to **(A)** naïve control or **(C,D)** D1 unless otherwise indicated.

Next, we compared the cell surface marker expression of ILC2s in steady state, in IL-33-mediated airway inflammation, as well as in HDM-induced allergic airway inflammation. Whereas IL-33 induced a robust expansion of ILC2s in BAL fluid, lung, MLN, and spleen, HDM exposure resulted in a significant but more modest ILC2 response in BAL fluid, lung, and MLN but not in spleen (Figure 2A), as previously reported (17, 22).

Stimulation by IL-33 resulted in high surface expression of CD25, ICOS, KLRG1, and T1/ST2 on BAL fluid ILC2s, which are markers frequently used to define these cells (1, 2). In contrast, HDM-activated ILC2s in BAL fluid displayed large phenotypic heterogeneity and showed substantially reduced expression levels of CD25, ICOS, KLRG1, and T1/ST2, which were even lower than on ILC2s from naïve mice. ILC2s in lung and MLN showed a similar picture in that surface expression of CD25, ICOS, and KLRG1 was lower on HDM-activated ILC2s than on IL-33-stimulated ILC2s. Cell surface expression of these three markers on spleen and bone marrow (BM) ILC2s were not different between mice with HDM-mediated inflammation and naïve mice, consistent with the expected localized effects of intranasal HDM exposure. T1/ST2 on ILC2s showed low expression in lung and spleen in both naïve mice and in mice with airway inflammation. On MLN and BM ILC2s the expression of T1/ST2 was upregulated following IL-33 but not following HDM exposure. IL-33-stimulated ILC2s in the BM were also high in KLRG1, typically expressed by inflammatory ILC2s in peripheral tissues, suggesting systemic ILC2 activation (2). In all compartments analyzed, the expression of CD117 and CD127 on ILC2s showed only minor differences between naïve and HDM-challenged mice. Whereas the effects of IL-33 on CD117 expression on ILC2s were modest, IL-33-stimulated ILC2s in lung, MLN, and spleen featured a detectable downregulation of CD127 expression (Figure 2B).

Next, we further investigated whether the ILC2 phenotype alters over time after exposure to IL-33. GATIR mice were challenged with IL-33 and sacrificed at several time points after the final challenge. As expected, eosinophil counts gradually decreased, but ILC2 numbers appeared to peak on day 3 (Figure 2C). ICOS and KLRG1 expression followed a similar pattern to eosinophils, while CD117 was upregulated at later time points. Remarkably, we found that while CD25 was initially highly expressed on IL-33-stimulated ILC2s, the expression was significantly decreased from day 3 after the final challenge and onward with similar trends in both BAL fluid and lung tissue (Figure 2D). Only minor changes were observed in T1/ST2 expression and Thy1.2 (CD90.2) was mildly upregulated by day 10 in a similar pattern as CD117 (Figure S3 in Supplementary Material). Furthermore, the percentage of ILC2s expressing proliferation marker Ki-67 significantly decreased at later time points, suggesting a decreased activation state (Figure S3 in Supplementary Material).

**Figure 3:**
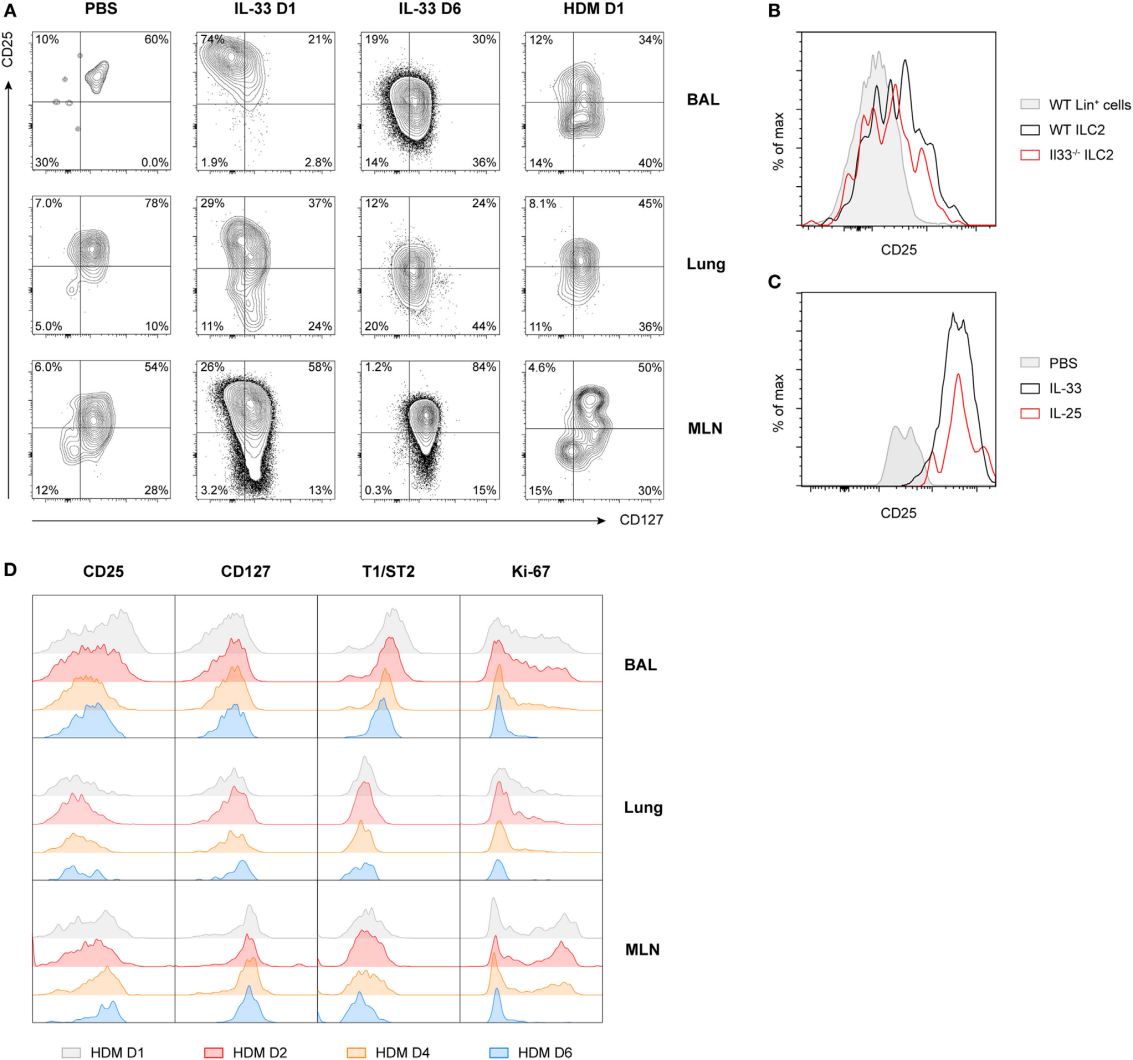
Expression of CD25 on group 2 innate lymphoid cells (ILC2s) is highly variable and time dependent. **(A)** Expression profiles of CD25 and CD127 on ILC2s from bronchoalveolar lavage (BAL) fluid, lung, and mediastinal lymph node (MLN) in PBS, IL-33 and house dust mite (HDM)-treated GATA3 IRES Reporter mice. **(B)** Flow cytometric analysis of CD25 expression on gated BAL fluid ILC2s in HDM-treated wild-type and *Il33^−/−^* mice, shown as histogram overlays, compared to control lineage marker positive cells. **(C)** Comparison of CD25 expression on gated BAL fluid ILC2s in PBS, IL-25, and IL-33-treated mice, shown as histogram overlays. **(D)** Expression profiles of CD25, CD127, T1/ST2, and Ki-67 over time on ILC2s from BAL fluid, lung, and MLN in HDM-treated mice. **(A–D)** Plots represent combined data using the concatenate option in FlowJo (*n* = 3–6), representative of two independent experiments.

The characteristic downregulation of CD25 expression revealed a type of ILC2 phenotypic heterogeneity that to the best of our knowledge has not been described previously. Particularly in BAL fluid, but also in lung and MLN, IL-33-stimulated ILC2s showed a CD25^high^CD127^low^ phenotype on day 1 after challenge while IL-33-exposed ILC2s on day 6 and HDM-activated ILC2s showed an inversed CD25^low^CD127^high^ phenotype (Figure 3A). When we specifically gated CD25^low^ ILC2s present in BAL fluid and lung from mice with HDM-driven inflammation, we found that these cells displayed low surface expression of ICOS, KLRG1, and T1/ST2 compared to gated CD25^high^ ILC2s. However, both CD25^high^ and CD25^low^ ILC2s highly expressed CD90.2 on the cell surface (data not shown). Additionally, CD25^high^ ILC2s present in HDM-driven and IL-33-driven inflammation showed differences as well, e.g., in surface CD127 expression in the lung and in ICOS expression in BAL fluid and lung (Figure S4 in Supplementary Material).

Consistent with our previous finding that ILC2 induction in BAL fluid in HDM-driven allergic airway inflammation is IL-33 independent (22), we noticed that the absence of IL-33 in HDM-exposed *Il33^−/−^* mice did not change CD25 expression on ILC2s compared to wild-type control mice (Figure 3B). Stimulation of ILC2s with IL-25 also did not result in altered CD25 expression compared to IL-33-stimulated mice (Figure 3C). However, CD25 expression did decrease further over time in HDM-challenged mice, in particular in the BAL fluid, although not as drastically as the kinetics seen in IL-33-challenged mice. Consistent with this finding, T1/ST2 and Ki-67 were also downregulated over time, while CD127 expression remained relatively stable (Figure 3D). We confirmed these findings in wild-type mice and found that, as expected, GATIR mice did not significantly differ from wild-type counterparts in both surface marker expression of ILC2s (Figure S5A in Supplementary Material) or cytokine production (Figure S5B in Supplementary Material) in BAL fluid, lung, or MLN.

Taken together, these findings show that in BAL fluid IL-33-stimulated ILC2s had an almost uniform CD25^+^CD127^+^T1/ST2^+^ICOS^+^KLRG1^+^ phenotype, while HDM-induced ILC2s displayed a heterogeneous surface phenotype characterized by substantially lower levels of CD25, ICOS, and KLRG1. In both models of airway inflammation, ILC2s exhibited variable expression of many surface markers commonly used to identify ILC2s with substantial differences across tissues and dependent on the time of analysis, suggesting that the microenvironment and the stage of inflammation are important factors in determining ILC2 phenotype.

### Both CD25^high^ and CD25^low^ ILC2s Have the Capacity to Produce Type 2 Cytokines

**Figure 4:**
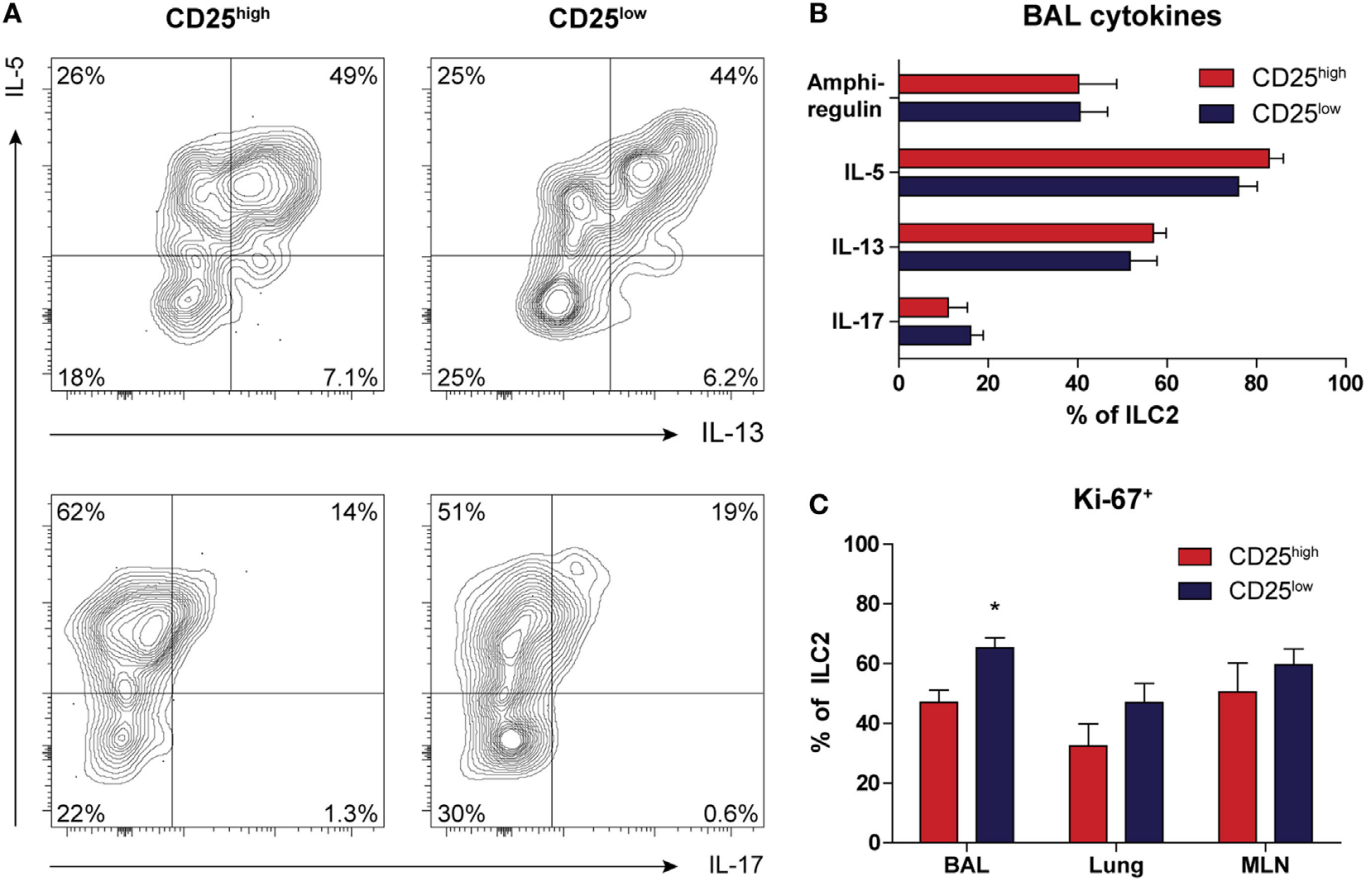
Both CD25^high^ and CD25^low^ group 2 innate lymphoid cells (ILC2s) in house dust mite (HDM)-driven airway inflammation have the capacity to produce type 2 cytokines. **(A)** Flow cytometric analysis of IL-5, IL-13, and IL-17 production in bronchoalveolar lavage (BAL) fluid by CD25^high^ and CD25^low^ ILC2s of HDM-treated mice. Plots represent combined data using the concatenate option in FlowJo (*n* = 6), representative of two independent experiments. **(B)** Proportions of cytokine producing ILC2s in the BAL fluid after HDM stimulation, stratified by CD25 expression. **(C)** Comparison of proliferative capacity between CD25^high^ and CD25^low^ ILC2s in BAL fluid, lung, and mediastinal lymph node (MLN) of mice after HDM treatment as indicated by Ki-67 expression. **(B,C)** Data are shown as mean + SEM of *n* = 6 mice per group of a single experiment and are representative of two independent experiments. *p ≤ 0.05 compared to CD25^high^ ILC2s unless otherwise indicated.

Next, we used intracellular flow cytometry following *in vitro* stimulation with PMA and ionomycin for 4 h to determine the capacity of ILC2 to produce type 2 cytokines. Both CD25^high^ and CD25^low^ ILC2s present in BAL fluid from HDM-challenged mice had a similar capacity to produce IL-5, IL-13, and amphiregulin (Figures 4A,B). The proportions of the BAL fluid ILC2s that were IL-5^+^ and IL-13^+^ in HDM-driven inflammation were ~75 and ~55%, respectively, which was significantly lower than the proportions observed upon IL-33 exposure (~95 and ~85%, respectively) (17). We found marginal levels of IL-4 production (data not shown), but IL-17 production was detectable in both CD25^high^ and CD25^low^ ILC2s, albeit at low levels (Figures 4A,B). In these experiments, we did not detect expression of IFN-γ. IL-17^+^ ILC2s were more clearly visible after IL-33 stimulation and decreased quite rapidly together with amphiregulin, in contrast to IL-5 and IL-13, which remained highly expressed (Figure S6A in Supplementary Material). Importantly, all IL-17-producing ILC2s also secreted IL-5, hinting at functional plasticity (Figure S6B in Supplementary Material). As analyzed by Ki-67 expression, CD25^low^ ILC2s were slightly more proliferative than CD25^high^ ILC2s in BAL fluid, but not in lung or MLN (Figure 4C).

In summary, low surface expression of CD25 did not appear to impact the capacity of ILC2 to produce cytokines. In addition, a proportion of CD25^high^ and CD25^low^ ILC2s were able to secrete IL-5 and IL-17 simultaneously, suggesting a hybrid ILC2/ILC3 function.

### CD25^low^ ILC2s in HDM-Treated Mice Are Reversed to a CD25^high^ Phenotype upon Subsequent Stimulation with IL-33

**Figure 5:**
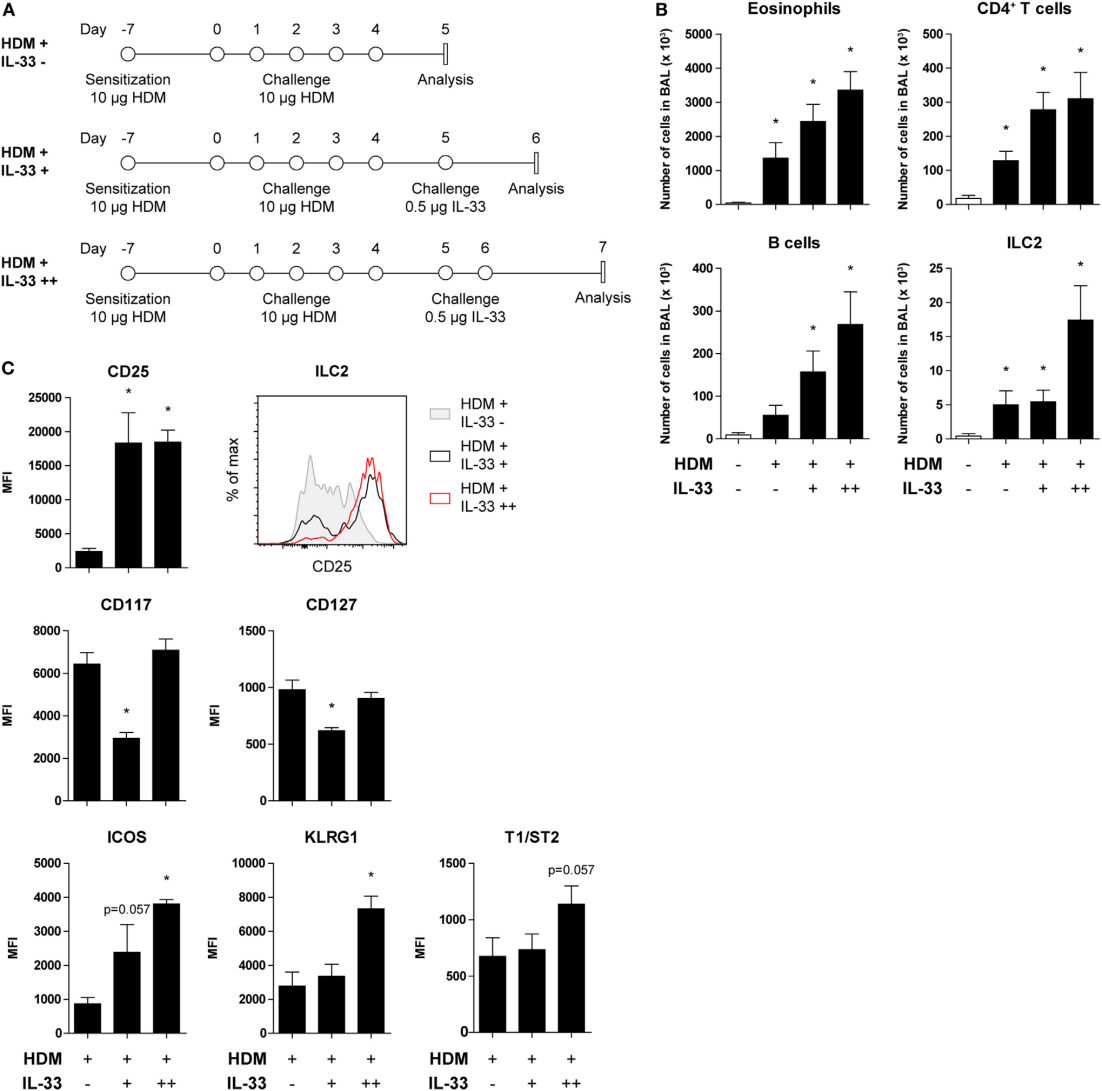
CD25 expression on house dust mite (HDM)-stimulated group 2 innate lymphoid cells (ILC2s) is restored by acute IL-33 exposure. **(A)** Schemes for administration of HDM with and without subsequent IL-33 exposure in GATA3 IRES Reporter mice. **(B)** Quantification of bronchoalveolar lavage (BAL) fluid eosinophils, CD4^+^ T cells, B cells, and ILC2s in animals treated with PBS, HDM alone or in combination with IL-33. **(C)** Surface marker expression profile of BAL fluid ILC2s. **(B,C)** Data are shown as mean + SEM of *n* = 4 mice per group of a single experiment. *p ≤ 0.05 compared to PBS control unless otherwise indicated. **(C)** Plot represents combined data using the concatenate option in FlowJo (*n* = 4).

Recent publications describing the plasticity and heterogeneity of ILC2s (32–35) prompted us to investigate the reversibility of the downregulation of CD25 on ILC2s as a result of HDM-induced allergic airway inflammation. GATIR mice were sensitized and challenged with HDM and subsequently treated with a single or double exposure to IL-33 (Figure 5A). Control mice were only treated with PBS. BAL fluid eosinophil, CD4^+^ T cell, and B cell counts were significantly elevated after HDM treatment and further exacerbated in a dose-dependent manner upon IL-33 exposure. ILC2 numbers did not increase with a single IL-33 treatment in HDM-exposed mice but required two consecutive IL-33 doses to expand (Figure 5B). However, CD25 expression on HDM-activated ILC2s was readily upregulated after a single IL-33 dose while numbers remained stable, indicating upregulation of CD25 expression on existing cells as opposed to influx or generation of new CD25^high^ ILC2s (Figure 5C). Other ILC2 activation markers, such as ICOS, KLRG1, and T1/ST2 were also upregulated, although less rapidly than CD25. Interestingly, the MFI values of CD117 and CD127 were initially decreased and returned to previously levels after a second IL-33 treatment, again highlighting the adaptability of ILC2 phenotype depending on the microenvironment (Figure 5C).

### Chronic HDM Exposure Predominantly Induces CD25^low^ ILC2s, Which Substantially Contribute to Type 2 Cytokine Production in the Airways

**Figure 6:**
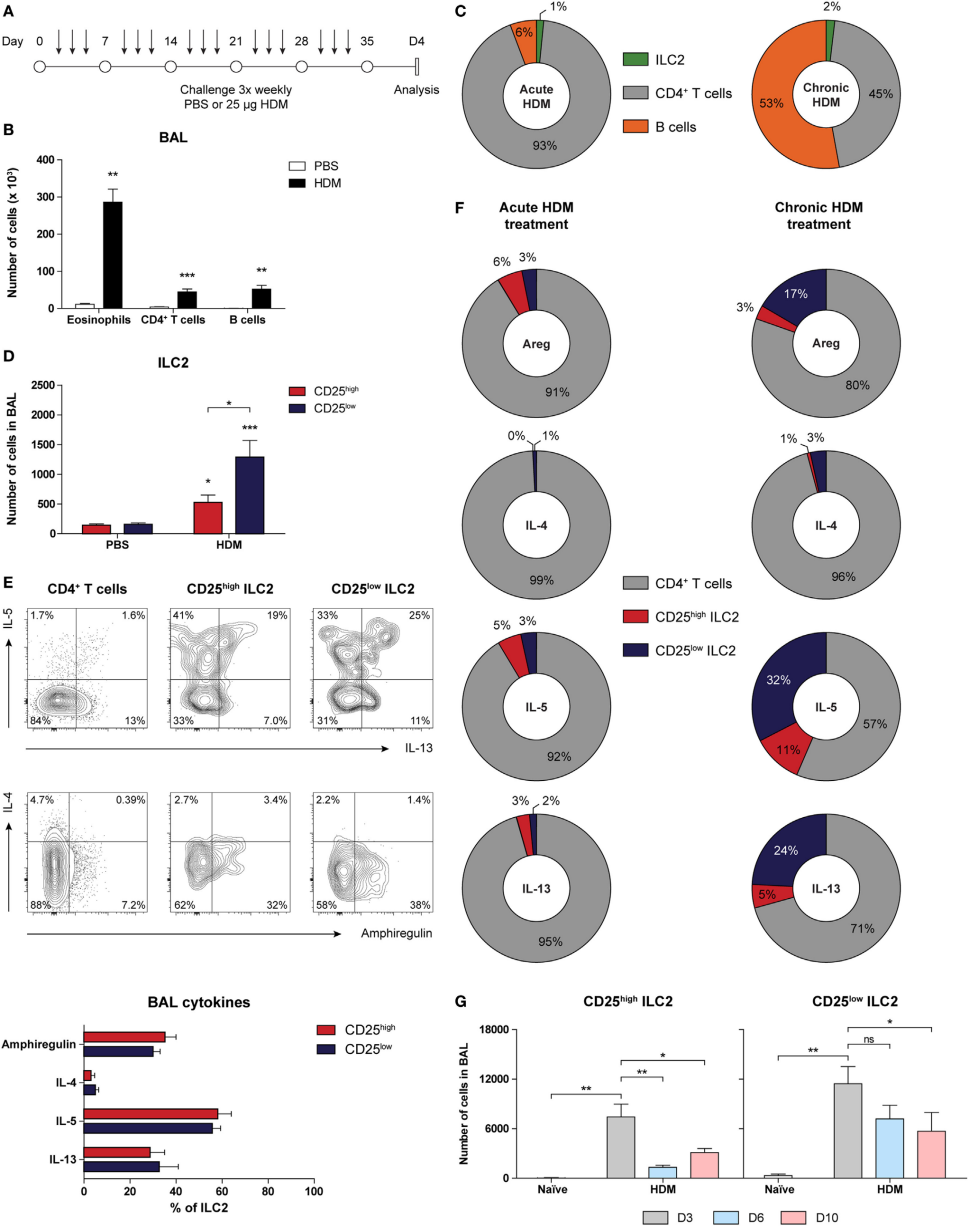
Group 2 innate lymphoid cells (ILC2s) with CD25^low^ phenotype are important cytokine producers in chronic house dust mite (HDM)-induced airway inflammation. **(A)** Scheme for intranasal HDM treatment of GATA3 IRES Reporter mice for induction of chronic allergic airway inflammation. **(B)** Quantification of eosinophils, CD4^+^ T cells, and B cells in the bronchoalveolar lavage (BAL) fluid of PBS and HDM-treated mice. **(C)** Relative contribution of CD4^+^ T cells, B cells, and ILC2s in the BAL fluid of acute and chronic HDM-treated mice. **(D)** Quantification of CD25^high^ and CD25^low^ ILC2s in the BAL fluid of PBS and HDM-treated mice. **(E)** Flow cytometric analysis and quantification of amphiregulin, IL-4, IL-5, and IL-13 production by CD4^+^ T cells, CD25^low^, and CD25^high^ ILC2s in the BAL fluid of HDM-treated mice. Plots represent combined data using the concatenate option in FlowJo (*n* = 7), representative of two independent experiments. **(F)** Relative contribution of CD4^+^ T cells, CD25^low^, and CD25^high^ ILC2s to the total number of amphiregulin (areg), IL-4, IL-5, and IL-13 producing cells in the BAL fluid of acute and chronic HDM-treated mice. **(G)** Numbers of CD25^high^ and CD25^low^ ILC2s in the BAL fluid after chronic HDM exposure at days 3, 6, and 10 after the final challenge. **(B,D,E,G)** Data are shown as mean + SEM of *n* = 5–7 mice per group of a single experiment and are representative of two independent experiments. *p ≤ 0.05, **p ≤ 0.01, ***p ≤ 0.001 compared to PBS control unless otherwise indicated.

Because ILC2s have been implicated in the maintenance of chronic asthma (53), we next investigated accumulation and phenotype of ILC2s in GATIR mice chronically exposed to HDM for a period of 5 weeks (Figure 6A). We have recently shown that chronic HDM exposure induces allergic airway inflammation, in addition to the formation of inducible bronchus-associated lymphoid tissue (iBALT) and tissue remodeling in the lungs (54). At day 4 after the final challenge, we detected an eosinophilic infiltrate in the BAL fluid, indicative of active inflammation (Figure 6B). Compared with our acute HDM-driven lung inflammation model, eosinophil numbers in BAL fluid were modest and the contribution of B cells was larger and in the same range as CD4^+^ T cells (Figure 6C). In comparison to PBS controls, the numbers of total ILC2s were significantly increased, of which the majority was CD25^low^ (Figure 6D) and both CD25^high^ and CD25^low^ populations of ILC2s in the BAL fluid expressed IL-5 and IL-13 (Figure 6E). Compared with the acute HDM-driven inflammation model, a more sizeable contribution of ILC2s to the total number of cytokine producing cells was observed in chronic HDM airway inflammation exposure, of which the CD25^low^ subset represented a larger proportion than the classic CD25^high^ subset (Figure 6F). Next, we investigated whether the longevity of CD25^high^ and CD25^low^ ILC2s was different in the resolution phase of chronic HDM-mediated allergic airway inflammation. We quantified CD25^high^ and CD25^low^ ILC2s at 3, 6, and 10 days after the last allergen challenge and found that total numbers of CD25^low^ ILC2s appeared to be more persistent than CD25^high^ ILC2s (Figure 6G).

Taken together, these findings show that ILC2s that are present in chronic HDM-driven allergic airway inflammation are prominent producers of IL-5 and IL-13 and have a CD25^low^ phenotype, particularly in the resolution phase of inflammation.

### Transcriptome Profiling Indicates That Acutely Activated ILC2s Are Functionally Different from ILC2s in Chronic Airway Inflammation

As outlined above, we found that the rapid and robust activation of ILC2s by IL-33 induces high levels of type 2 cytokine production by these cells (17) and an almost uniform CD25^+^CD127^+^T1/ST2^+^ ICOS^+^KLRG1^+^ phenotype. In contrast, CD25^low^ ILC2s, exhibiting lower surface expression of T1/ST2, ICOS, and KLRG1 and a more modest cytokine production capacity, dominated in our chronic HDM airway inflammation model.

**Figure 7:**
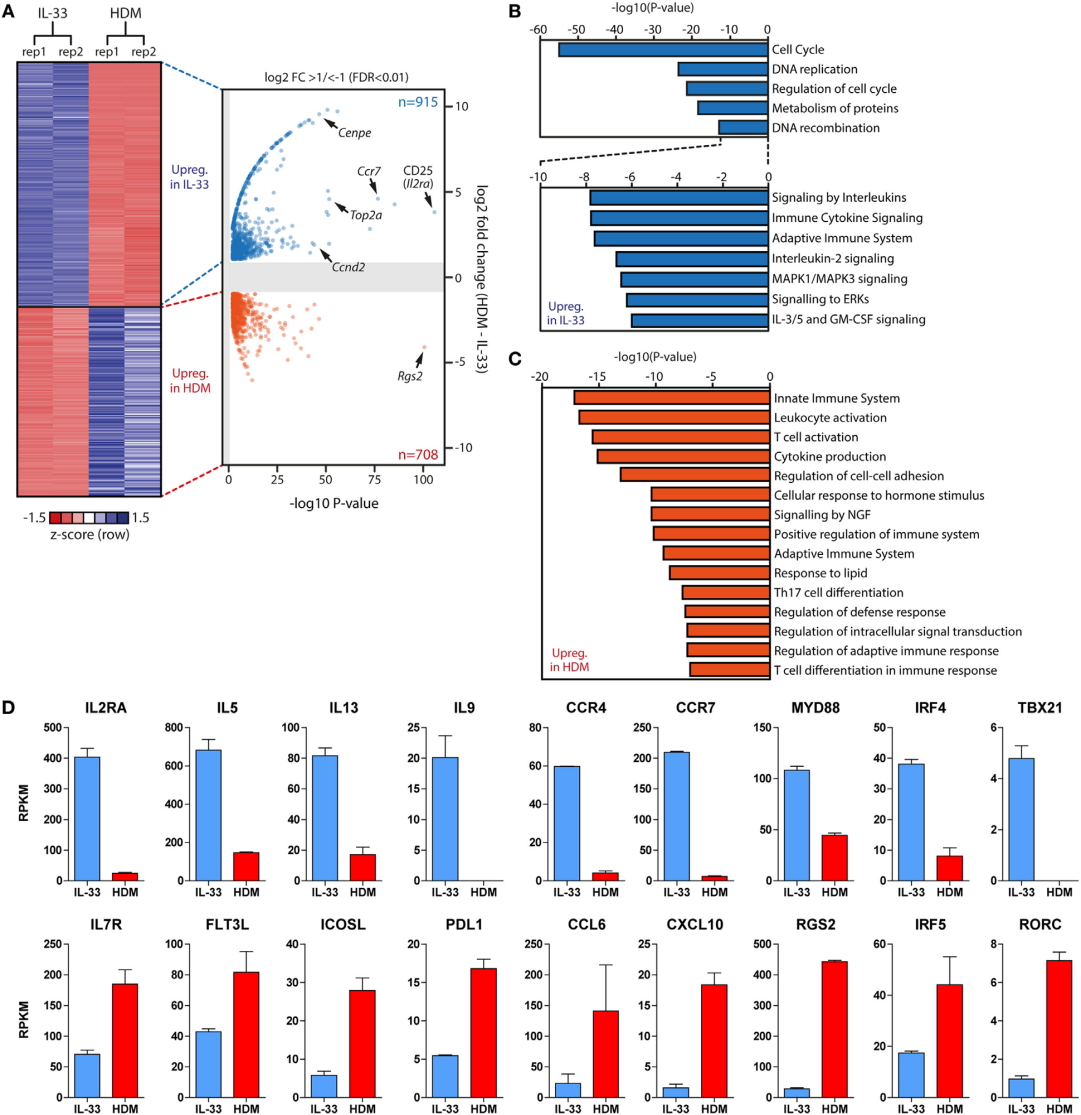
Gene expression signatures of group 2 innate lymphoid cells (ILC2s) are dependent on type and duration of stimulus. **(A)** Heat map and volcano plot of differentially expressed genes between IL-33 (*n* = 915) and chronic house dust mite (HDM)-stimulated (*n* = 708) ILC2s derived from bronchoalveolar lavage fluid 1 day after the final challenge. **(B,C)** Metascape pathway analysis of genes upregulated in **(B)** IL-33-activated and **(C)** chronic HDM-stimulated ILC2s. **(D)** Comparison of Reads Per Kilobase Millionvalues of selected differentially expressed genes in IL-33-activated (*blue*) and chronic HDM-stimulated (*red*) ILC2s.

To explore differences between acutely and chronically activated ILC2 populations in an unbiased and comprehensive fashion, we FACS-sorted BAL fluid ILC2s from GATIR mice 1 day after the final challenge following IL-33 or chronic HDM exposure, respectively, and analyzed gene expression in these cell fractions by RNA-Seq. We identified a total number of 1,623 differentially expressed genes, of which 915 were upregulated in IL-33-stimulated ILC2s and 708 in chronic HDM-stimulated ILC2s. Indeed, we found that the most significantly upregulated gene in IL-33-stimulated ILC2s was *Il2ra* encoding CD25 (Figure 7A), confirming our flow cytometry data. Pathway analysis of these genes indicated that processes related to cell proliferation and division (*Cenpe, Top2a, Ccnd2*) are highly active after IL-33 stimulation (Figures 7A,B). In contrast, HDM-exposed ILC2s showed a transcriptional signature associated with regulation of the immune system and immune cell activation, including “innate immune system” genes, such as *Cd33, Ccr2, Ctsd, and Ctss* (encoding Cathepsins D and S) and *Csf2* (encoding GM-CSF) (Figure 7C). Interestingly, chronic HDM exposure generated ILC2s with increased expression of genes implicated in the modulation of T cell activity (*Flt3l, Icosl, Pdl1*) and chemo-attraction of T and B cells (*Ccl6, Cxcl10*) (Figure 7D). Single nucleotide polymorphisms in *Rgs2*, and IRF5 activity have been associated with asthma (55, 56) and our data highlighted upregulated transcription of these genes in HDM-stimulated ILC2s (Figures 7A,D). Acutely, IL-33-activated ILC2s produced high levels of cytokine (*Il5, Il13, Il9*) and tissue-migratory chemokine receptor (*Ccr4, Ccr7*) genes, indicating an activated effector cell phenotype. Furthermore, mediators of innate signals were also prominent, including the TLR-signaling protein Myd88 and IRF4, which is known to respond to IL-33 and TSLP (57). Surprisingly, IL-33-activated ILC2s showed low but detectable *Tbx21* (encoding T-bet) transcripts, whereas we detected increased *Rorc* (encoding RORγt) levels in HDM-activated ILC2s, indicative of ILC2 plasticity toward group 1 and group 3 ILCs, respectively (Figure 7D).

Taken together, these data suggest distinct roles of ILC2s dependent on the type and the duration of stimulus. While acutely activated ILC2s by IL-33 displayed a strong effector cell phenotype, chronically HDM-stimulated ILC2s appear to regulate and assist adaptive immunity.

### ILC2s Are Situated Below the Lung Epithelium and Inside Cellular Infiltrates Induced by Acute and Chronic Airway Inflammation

**Figure 8:**
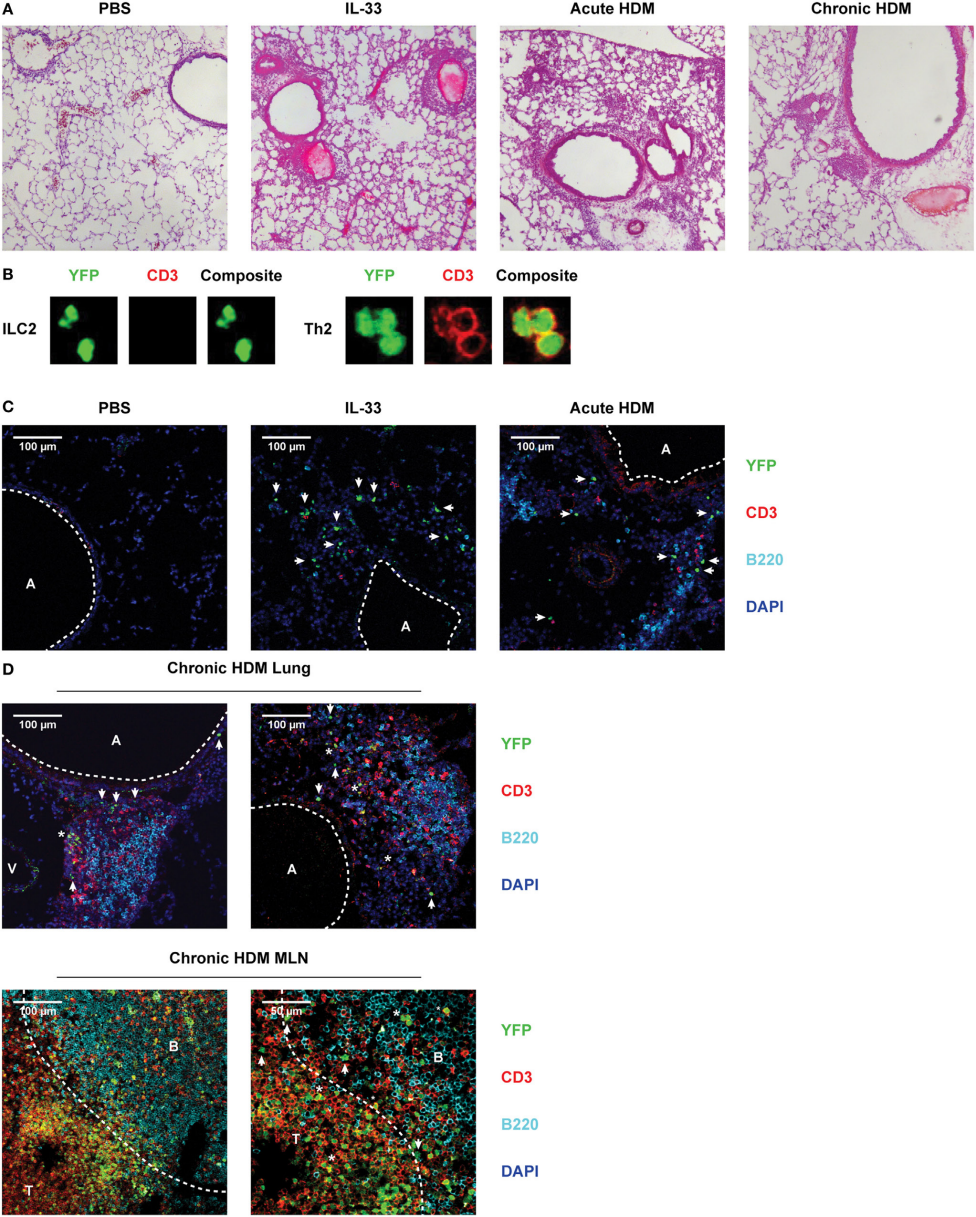
Pulmonary group 2 innate lymphoid cells (ILC2s) are located within cellular infiltrates underneath the lung epithelium after IL-33, acute and chronic house dust mite (HDM) exposure. **(A)** Hematoxylin and eosin stained cryosections of lungs from PBS, IL-33, acute HDM and chronic HDM-treated GATA3 IRES Reporter (GATIR) mice. **(B)** Discrimination between ILC2s and Th2 cells by counterstain with CD3 and detection by confocal microscopy. **(C)** Lung cryosections from PBS, IL-33, and acute HDM-treated GATIR mice counterstained with CD3 and B220. **(D)** Lung and mediastinal lymph node (MLN) cryosections from chronic HDM-treated GATIR mice counterstained with CD3 and B220. In the confocal microscopy conditions, GATA3^low^ cells (such as non-Th2 CD4^+^ T cells and basophils) are not YFP^+^. It is of note that in chronic HDM-driven airway inflammation, only ~10% of all CD4^+^ T cells in the lung consist of GATA3^high^ Th2 cells (54). [A] indicates airway lumen. [V] indicates lumen of blood vessel. ILC2s are indicated by [arrows] and [*] indicates Th2 cells. [B] and [T] indicate B and T cell zones, respectively, separated by the dashed line. **(A–D)** Images are representative of two independent experiments.

The GATIR knock-in allowed us to visualize Th2 cells and ILC2s as YFP^+^ cells *in situ* using confocal microscopy. Our RNA-Seq data suggest contrasting functions between rapidly and chronically activated ILC2s, thus we examined whether IL-33 and HDM-stimulated ILC2s differed in localization in the lungs. Exposure to IL-33, acute and chronic HDM according to the protocols described in Figures 1A,B and 6A generated lung inflammation outlined by perivascular and peribronchial cellular infiltrates and thickened alveolar walls compared to PBS treated mice (Figure 8A). Lung cryosections were counterstained with a combination of CD3 and B220 to label T and B cells, respectively. YFP^+^ ILC2s were readily discriminated from YFP^+^ Th2 cells by the absence of positivity for CD3 counterstaining (Figure 8B).

Following IL-33 stimulation many CD3^−^YFP^+^ ILC2s were identified in cellular infiltrates directly underneath the lung epithelium, with T and B cells being largely absent (Figure 8C), corroborating previously reported data and our flow cytometric data (28, 29). *In situ* hybridization of GATA3 revealed signals in similar locations as YFP^+^ cells (Figure S7 in Supplementary Material), supporting that YFP signals reflect *Gata3* transcription. In contrast, in acute HDM-induced airway inflammation T and B cells were present in higher numbers in the cellular infiltrates. Hereby, also CD3^−^YFP^+^ ILC2s were identified, although less abundantly present than after IL-33 stimulation (Figure 8C). The ILC2s were not present in clusters and we did not find evidence for frequent interaction with B or T cells.

Chronically HDM-treated GATIR mice displayed cellular infiltrates surrounding blood vessels and near the airways in similar locations as acute lung inflammation models. However, the infiltrates appeared denser in a hematoxylin and eosin stain (Figure 8A) and confocal microscopy revealed a well-defined organization within these infiltrates (Figure 8D). The infiltrates were composed of separated B and T cell zones with striking similarity to iBALT structures found in influenza-infected lungs (58). CD3^−^YFP^+^ ILC2s were located at two distinct sites: in the submucosa close to epithelial cells, comparable to their location in IL-33-induced airway inflammation, as well as within the cellular infiltrates close to the lung epithelium and in proximity of T cell clusters (Figure 8D). Interestingly, B cell areas were devoid of ILC2s suggesting preferential interaction of ILC2s with T cells. Neither in the submucosa nor within the cellular infiltrates did we find evidence for clustering or organization of ILC2s. Abundant numbers of Th2 cells, identified as CD3^+^YFP^+^ cells were found in the enlarged MLNs of chronically HDM-treated animals; CD3^−^YFP^+^ ILC2s were present near the border of the B and T cell zones (Figure 8D).

In summary, these data indicate that ILC2s accumulate in cellular infiltrates underneath the lung epithelium after activation. In chronic HDM-driven inflammation, ILC2s are mainly located in the submucosa close to epithelial cells, but are also found inside organized cellular infiltrates in close proximity to both epithelial cells and T cells, but not B cells.

## Discussion

In type 2 airway inflammation, ILC2s act as an early innate source of IL-5 and IL-13 when strong allergenic stimuli induce epithelial pro-inflammatory cytokines, including IL-33, that have the capacity to directly activate ILC2s independently of T cells (1, 2). However, because we previously found that in HDM-mediated allergic airway inflammation ILC2s are activated by different T cell-dependent pathways and IL-33 is not required for the HDM-driven induction of ILC2s in the BAL fluid (22), we hypothesized that ILC2 phenotype, function, and localization in HDM- and IL-33-driven inflammation may be different.

When using GATIR mice in an acute HDM-driven airway inflammation model, we detected the induction of a much more heterogeneous ILC2 population in BAL fluid, lungs, and draining lymph nodes than upon robust IL-33-induced ILC2 activation. We found that a large proportion of the ILC2s expressed low levels of CD25, and that these CD25^low^ cells were generally low for KLRG1, T1/ST2, and ICOS on the cell surface as well, although we observed compartmental differences (Figure 2B; Figure S4 in Supplementary Material). Nevertheless, they displayed a type 2 cytokine profile that was similar to their classic CD25^high^ counterparts. The CD25^low^ ILC2s in HDM-treated mice could be reverted back to a CD25^high^ phenotype by subsequent exposure to IL-33 and ILC2s lost high CD25 expression by day 3 after IL-33 treatment, demonstrating the adaptability of ILC2s. We also observed that the levels of cell surface marker expression are highly dynamic over time, as, e.g., shown by our analysis of CD25, CD117, ICOS, and KLRG1 surface expression at different time points after the last IL-33 exposure (Figure 2C).

Our IL-33 and HDM-treatment models are similar in terms of the activation period of ILC2s and the timing of the analysis: 1 day after the last challenge (Figures 1A,B). In this context, it is of note that we have previously shown that in the HDM-driven model ILC2s are only activated in the allergen challenge phase when memory T cells are present and not upon sensitization, even when a single dose of 100 µg of HDM is given (22). It is, therefore, conceivable that certain differences in ILC2 phenotype between IL-33- and HDM-stimulated ILC2s are related to changes in cell–cell interactions or cytokine environment, given that activation of ILC2s by IL-33 and HDM is T cell-independent and T cell-dependent, respectively (17, 22). Collectively, our findings show that the cell surface marker composition of ILC2s is highly dynamic: markers follow a specific kinetic pattern after activation and are dependent on the mechanisms by which these cells are activated, e.g., a requirement of T cells for their activation.

It is of note that quite some heterogeneity in the expression levels of cell surface molecules, including CD25, CD69, CD117, ICOS, and T1/ST2 on resident ILC2s in the lungs of naïve mice has been observed among published studies, which is likely due to differences in the experimental models and housing conditions of the mice (59). On the other hand, our finding of, e.g., lower expression of T1/ST2 on lung ILC2s than on BAL ILC2s upon IL-33 or HDM-stimulation parallels reported observations in a *Nippostrongylus brasiliensis* infection model (60).

In chronic HDM-driven inflammation the CD25^low^ ILC2s outnumbered CD25^high^ ILC2s, remained elevated at least 10 days after the last antigen challenge in the resolution phase of inflammation and produced substantial amounts of IL-5 and IL-13, thus contributing significantly to the total Th2 cytokine production. Transcriptome comparisons of BAL fluid ILC2 populations from mice with acute IL-33-driven and chronic HDM-driven allergic airway inflammation revealed distinct gene expression profiles. Rapid and robust activation of ILC2s by IL-33 resulted in elevated transcript levels of genes involved in cellular proliferation and cytokine production. In contrast, in chronic HDM-mediated airway inflammation, ILC2s showed a transcriptional signature consistent with a capacity to modulate adaptive immunity by interacting with B and T cells. Visualization of ILC2s by confocal microscopy showed their submucosal accumulation upon IL-33 exposure and their presence in cellular infiltrates upon HDM exposure. Chronic exposure to HDM was associated with the formation of organized cellular clusters with defined T and B cells zones where ILC2s were located in close proximity to epithelial cells and T cells but not B cells and did not appear to form clusters.

Development and function of ILC2s critically depends on high levels of unperturbed GATA3 expression (39–41). To facilitate identification of ILC2 populations, potentially differing with respect to the expression levels of commonly used ILC2-associated surface markers, e.g., CD25 or KLRG1, we employed a novel strain of *Gata3* reporter mice, termed GATIR, carrying an IRES-YFP reporter construct within the 3′ untranslated region of the endogenous *Gata3* alleles (Tata Nageswara Rao and Hans Jörg Fehling, manuscript in preparation). Knock-in reporter mice are not without pitfalls and should not perturb the target gene expression itself. Therefore, we provided evidence that the presence of the GATIR reporter affected neither GATA3 protein levels (by intracellular flow cytometry with GATA3-specific antibodies), nor ILC2 surface marker profile or cytokine production. Although the IRES-driven reporter construct monitors gene expression at the transcript level, our intracellular flow cytometric analysis with GATA3-specific antibodies revealed a strong correlation between YFP signals and GATA3 protein in ILC2s and T cells.

Group 2 innate lymphoid cells were considered to be relatively homogeneous and less plastic compared to the other ILC family members. This point of view may have been triggered by the use of robust ILC2 induction models, such as administration of IL-25, IL-33, papain, and *Alternaria* to study these cells in the context of allergic asthma or dermatitis (15, 16, 18, 19, 61, 62). Our finding that in allergic airway inflammation ILC2s are a phenotypically and functionally more heterogeneous and phenotypically dynamic cell population than previously thought adds to the modified picture of ILC2s that includes their functional plasticity driven by IL-12, IL-18, or viral infection (32–35). In line with this notion, we detected simultaneous IL-5 and IL-17 production in HDM- and in particular IL-33-stimulated ILC2s and our results may support that ILC2s can upregulate T-bet or RORγt mRNA levels in a stimulus-specific manner. Phenotypical heterogeneity within the ILC2s induced in type 2 inflammation together with their functional plasticity allows ILC2s to adapt to changes in their microenvironment. Therefore, it is conceivable that these characteristics allow ILC2s to be involved in different human asthma endotypes, ranging from classic eosinophilic allergic airway inflammation to non-allergic airway hyperreactivity, as well as in infection-associated exacerbations.

By investigating the expression of several markers commonly used to define ILC2s, we identified a yet undescribed CD25^low^ ILC2 surface phenotype. CD25 appeared to correlate well with the expression of T1/ST2 and in particular KLRG1, which is known as a marker for mature and inflammatory ILC2s (39, 63). This might suggest that the CD25^low^ ILC2 population reflects an immature and non-inflammatory resident ILC2 population. However, in contrast to ILC2 progenitors in the BM, these CD25^low^ ILC2s are able to produce effector cytokines and expand upon stimulation with HDM, which would argue against their immature nature (39, 64). In further support of this concept, we found that CD25^low^ ILC2s (i) are typically present after IL-33 exposure when the total numbers of ILC2s in the BAL are highest (Figures 2C,D) and (ii) show prolonged cytokine production and contribute to the type 2 cytokine environment in our chronic HDM-driven allergic airway inflammation model. Indeed, the more pronounced role of ILC2s in chronic inflammation could be attributed to the acquisition of “memory-like” properties, as recently described (65). Such a memory phenotype allows experienced ILC2s to persist for extended periods of time and respond more potently than naïve ILC2s. Cytokine signaling through the common gamma chain is required for the survival of ILC2s and it has been demonstrated that IL-7 and in particular IL-2 synergistically augment ILC2 expansion in the presence of IL-33, which in turn promotes expression of CD25 in both adipose and lung tissue, thus forming a positive feedback loop by modulating sensitivity to IL-2 (6, 17, 66–69). Accordingly, our study also demonstrates upregulation of CD25 after IL-33 exposure of HDM-experienced ILC2s. The source of IL-2 has earlier been proposed to be T cell-derived, but recent data using IL-2 fate reporter mice has revealed that pulmonary ILC3 could be an innate producer of IL-2 (25, 69).

We found that high CD25 expression on the cell surface of IL-33-activated ILC2s was associated with a decrease in CD127 expression and conversely that CD25^high^ and CD25^low^ HDM-activated ILC2 showed persistent CD127 expression. This may imply that the CD25^low^ ILC2s are more reliant on IL-7 for their survival and function. Therefore, it could be speculated that these ILC2s can partly escape cross-regulation by Tregs, which are reported to control ILC2 responses by competition for IL-2 uptake (70). In a recent study, coculture of ILC2s with Tregs was shown to downregulate ICOS and CD25 expression (71). It is conceivable that CD25^low^ ILC2s in HDM-mediated airway inflammation represent ILC2s that have interacted with Tregs *in vivo*, because IL-5 and IL-13 levels are substantially decreased in ILC2s cocultured *in vitro* with Tregs (71), and our expression analyses demonstrate that the CD25^low^ ILC2s in HDM-driven inflammation have lower levels of cytokine transcripts than IL-33 activated ILC2s. However, it cannot be formally excluded that CD25^low^ IlC2s are cells that just received an IL-2 stimulus leading to downregulation of the receptor. Based on our findings in *Il33*^−/−^ mice, we conclude that IL-33 is unlikely to play a key role in the regulation of CD25 in HDM-mediated allergic airway inflammation. The combination of signals that specifically triggers CD25 up- and downregulation on ILC2s remains to be elucidated. Although expression of CD25 has been reported in human ILC2s from skin biopsies, adipose tissue and *in vitro* cultured ILC2s derived from peripheral blood, it is unknown whether pulmonary ILC2s also express this marker and its role in disease pathology has not been elucidated (26, 35, 61, 62).

Mice that were chronically exposed to HDM displayed well-organized and dense cellular clusters in the lungs reminiscent of iBALT structures seen after influenza infection (58). These structures harbored clear B and T cell zones and we observed ILC2s in proximity to T cells and not B cells. Indeed, ILC2s can express molecules that allow them to interact with T cells such as MHC class II, CD86, and ICOS/ICOS-L in addition to their cytokine repertoire (25–27). In the lungs, their positioning would allow them to act as an intermediate messenger to shuttle signals from the epithelium to T cells. For example, activation of ILC2s by epithelial signals can help reinforce a Th2 phenotype through direct cell contact. Conversely, CD25^high^ ILC2s may become more activated in the presence of T cell-derived IL-2 and promote alternatively activated macrophages through IL-13, which in turn assist with repair of the epithelium and resolution of inflammation. In the lymph nodes, we found that ILC2s were situated along the border of B and T cells, an optimal site to communicate with both cell types. In this context, our RNA-Seq data support the notion that ILC2s have the ability to interact with adaptive immune cells and revealed that HDM-stimulated ILC2s showed robust expression of FLT3L, ICOS-L, and PDL1, which would allow ILC2s to regulate B and T cell functions. Additionally, ILC2-derived factors such as IL-5 can enhance Ig production (72).

In summary, in addition to the reported plasticity of ILC2 toward other ILC subsets, we found that the ILC2 phenotype is highly dynamic. This phenotype is dependent on the mode of cellular activation, changes over time during stimulation, exhibits differences across tissues, and is reversible. In particular, we identified previously undescribed ILC2s with a GATA3^+^CD25^low^ phenotype and a type 2 cytokine profile indistinguishable from their classic GATA3^+^CD25^high^ counterparts. These CD25^low^ ILC2s contributed substantially to type 2 cytokine production and are able to readily revert to a CD25^high^ phenotype upon stimulation with IL-33. Although it cannot be excluded that CD25^high^ and CD25^low^ ILC2s represent separate subsets, the observed changes of CD25, KLRG1, ICOS, and T1/ST2 over time and differences between IL-33-activated and HDM-driven CD25^high^ ILC2s point to a different activation status of a single ILC2 type. Our data suggest a more complex ILC2 phenotype than is currently appreciated. Hereby, distinct functional capabilities of ILC2s, including responsiveness to IL-2, IL-33, or ICOS-L, are tissue-specific and dependent on the type and duration of the stimulus used. The observed phenotypic heterogeneity should, however, not hamper the identification and quantification of ILC2s as long as GATA3, which is invariably expressed by ILC2s, Sca-1, and CD90 are included in flow cytometric analysis strategies.

## Ethics Statement

All animal care and experimental procedures were performed in accordance to guidelines approved by the Erasmus MC Animal Ethics Committee.

## Author Contributions

BL, RS, MDB, ML, DB, MB, AK, and IB, performed the experiments; HV and MK provided methodologies (chronic HDM mouse model); WI provided methodologies (RNA-Seq); TR and HF provided essential materials (GATIR mouse). BL and RS analyzed the data. BL, RS, and RH conceived the study and wrote the manuscript.

## Conflict of Interest Statement

The authors declare that the research was conducted in the absence of any commercial or financial relationships that could be construed as a potential conflict of interest.

## Footnotes

^1^http://metascape.org.

^2^http://fiji.sc/.

## Abbreviations

BAL, bronchoalveolar lavage; BM, bone marrow; CLP, common lymphoid progenitor; HDM, house dust mite; iBALT, inducible bronchus-associated lymphoid tissue; ILC, innate lymphoid cell; ILC1, group 1 innate lymphoid cell; ILC2, group 2 innate lymphoid cell; ILC3, group 3 innate lymphoid cell; MLN, mediastinal lymph node; OVA, ovalbumin; PMA, phorbol 12-myristate 13-acetate; RNA-Seq, RNA sequencing; Th, T helper; Th2, T helper 2; Treg, T regulatory; YFP, yellow fluorescent protein.
