# Supplementary material for: Group 2 Innate Lymphoid Cells Exhibit a Dynamic Phenotype in Allergic Airway Inflammation
Source: Front Immunol. 2017 Dec 1;8:1684. doi: 10.3389/fimmu.2017.01684 (PMC5716969; doi:10.3389/fimmu.2017.01684)
Supplement: Supplementary file 1 [file data_sheet_1.docx]

**Supplementary file to:**

**Group 2 innate lymphoid cells exhibit a dynamic phenotype in allergic airway inflammation**

Bobby W.S. Li^1^, Ralph Stadhouders^1^, Marjolein J.W. de Bruijn^1^, Melanie Lukkes^1^, Dior M.J.M. Beerens^1^, Maarten D. Brem^1^, Alex KleinJan^1^, Ingrid Bergen^1^, Heleen Vroman^1^, Mirjam Kool^1^, Wilfred F.J. van IJcken^2^, Tata Nageswara Rao^3*^, Hans Jörg Fehling^3^ and Rudi W. Hendriks^1^

^1^Department of Pulmonary Medicine and ^2^Center for Biomics, Erasmus MC Rotterdam, Rotterdam, the Netherlands, ^3^Institute of Immunology, University Clinics Ulm, Ulm, Germany

^*^Current address: Department of Biomedicine, Experimental Hematology, University Hospital Basel, Basel, Switzerland

**
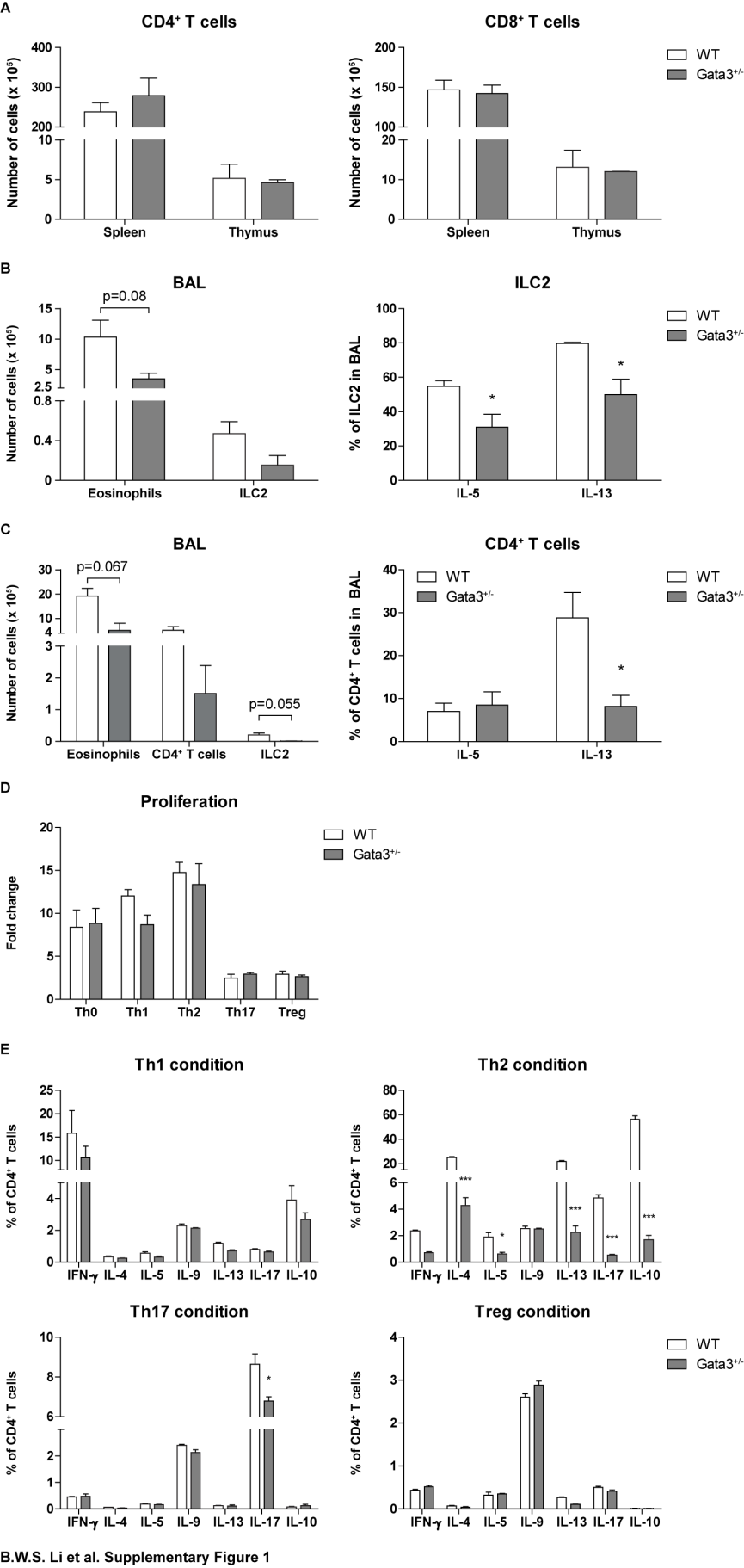
Figure S1. Reduction of GATA3 levels impairs ILC2 and Th2 induction and cytokine production.**

(A) Quantification of CD4^+^ and CD8^+^ T cells in the spleen and thymus of naïve wildtype (WT) and *Gata3^+/-^* mice.

(B) Quantification of eosinophils, ILC2s and ILC2 cytokine production in broncho-alveolar lavage (BAL) fluid of WT and *Gata3^+/-^* mice stimulated with IL-33.

(C) Quantification of eosinophils, CD4^+^ T cells, ILC2s and CD4^+^ T cell cytokine production in BAL fluid of WT and *Gata3^+/-^* mice stimulated with house dust mite (HDM).

(D) *In vitro* proliferation of naïve CD4^+^ T cells from WT and *Gata3^+/-^* mice cultured under Th0, Th1, Th2, Th17 and Treg polarizing conditions.

(E) Cytokine profiles of CD4^+^ T cells from WT and *Gata3^+/-^* mice cultured under the indicated polarizing conditions.

(A-C) Data are shown as mean + SEM of n = 3-5 mice per group of a single experiment and are representative of two independent experiments.

(D, E) Data are shown as mean + SEM of n = 3 mice per group of a single experiment and are representative of three independent experiments.

* p ≤ 0.05, ** p ≤ 0.01, *** p ≤ 0.001 compared to WT control unless otherwise indicated.

**
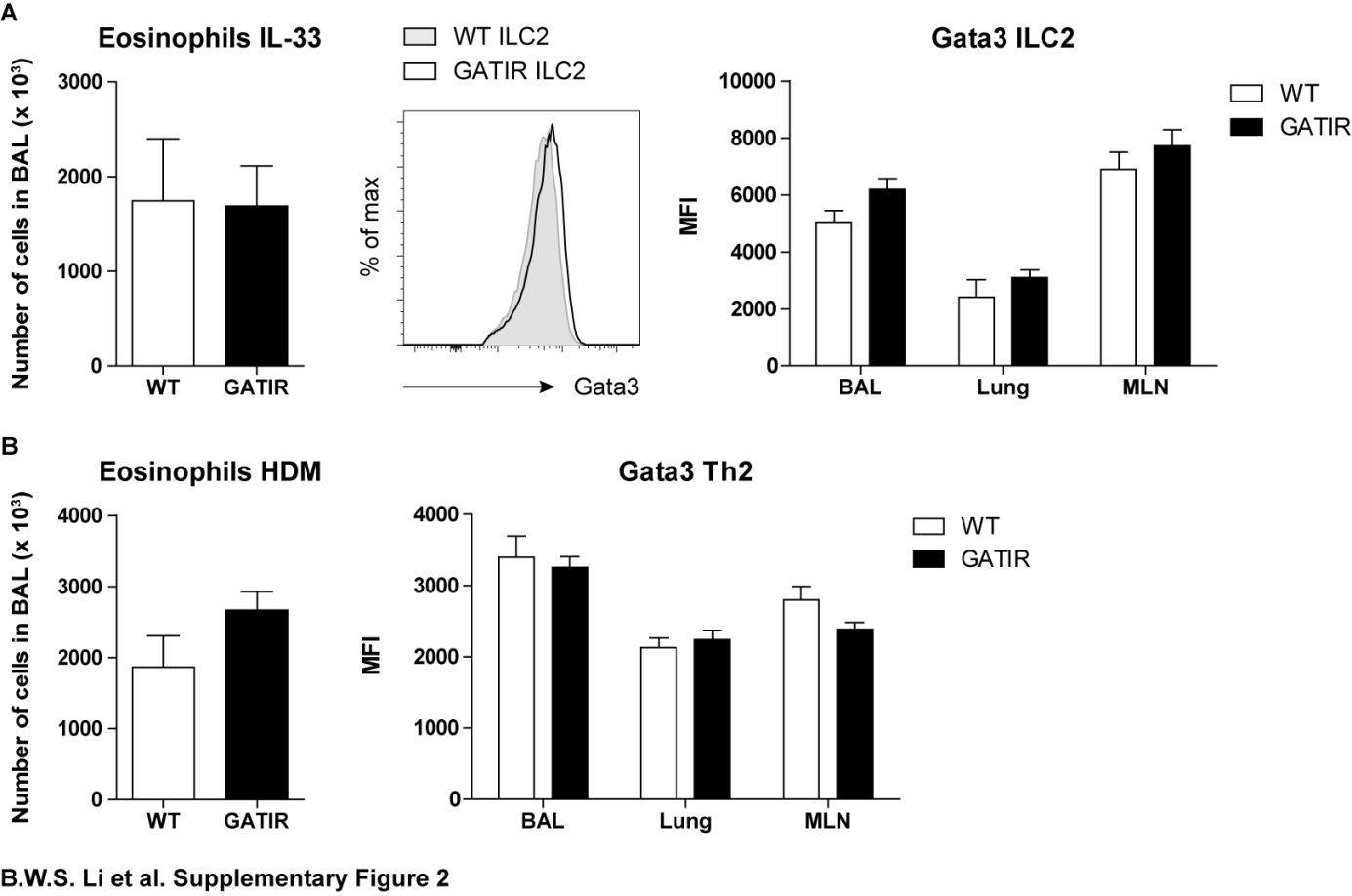
**

**Figure S2. GATIR knock-in does not affect GATA3 expression in ILC2s and T cells.**

(A) Number of BAL fluid eosinophils (*left*) and histogram overlay of intracellular GATA3 expression, as determined by flow cytometry in ILC2s from BAL fluid (*middle*) and comparison of GATA3 mean fluorescence intensity (MFI) values in ILC2s between IL-33-treated GATIR mice and WT controls (*right*). Plot represents combined data using the concatenate option in FlowJo (n = 3-5), representative of two independent experiments.

(B) Number of BAL fluid eosinophils (*left*) and comparison of GATA3 MFI values in CD4^+^ T cells between HDM-treated GATIR mice and WT controls (*right*). (A, B) Data are shown as mean + SEM of n = 3-5 mice per group of a single experiment and are representative of two independent experiments.


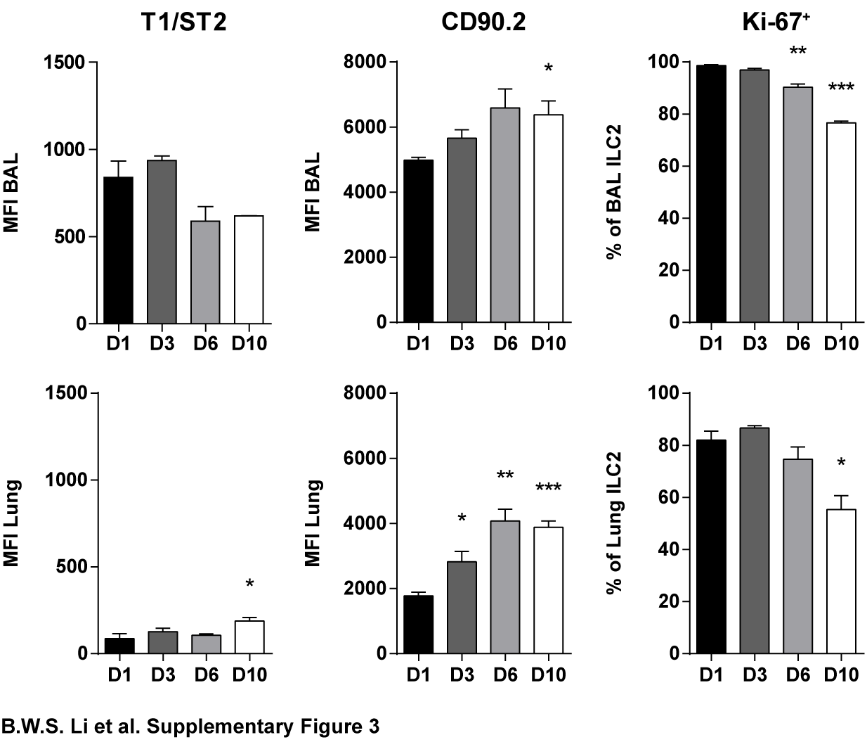


**Figure S3. Expression of T1/ST2, CD90.2 and Ki-67 in IL-33-stimulated ILC2s.**

MFI values of T1/ST2 and CD90.2 in ILC2s and percentage of Ki-67 expressing ILC2s in BAL fluid and lung at the indicated time points after IL-33 stimulation. Data are shown as mean + SEM of n = 3 mice per group of a single experiment and are representative of two independent experiments. * p ≤ 0.05, ** p ≤ 0.01, *** p ≤ 0.001 compared to D1 unless otherwise indicated.

**
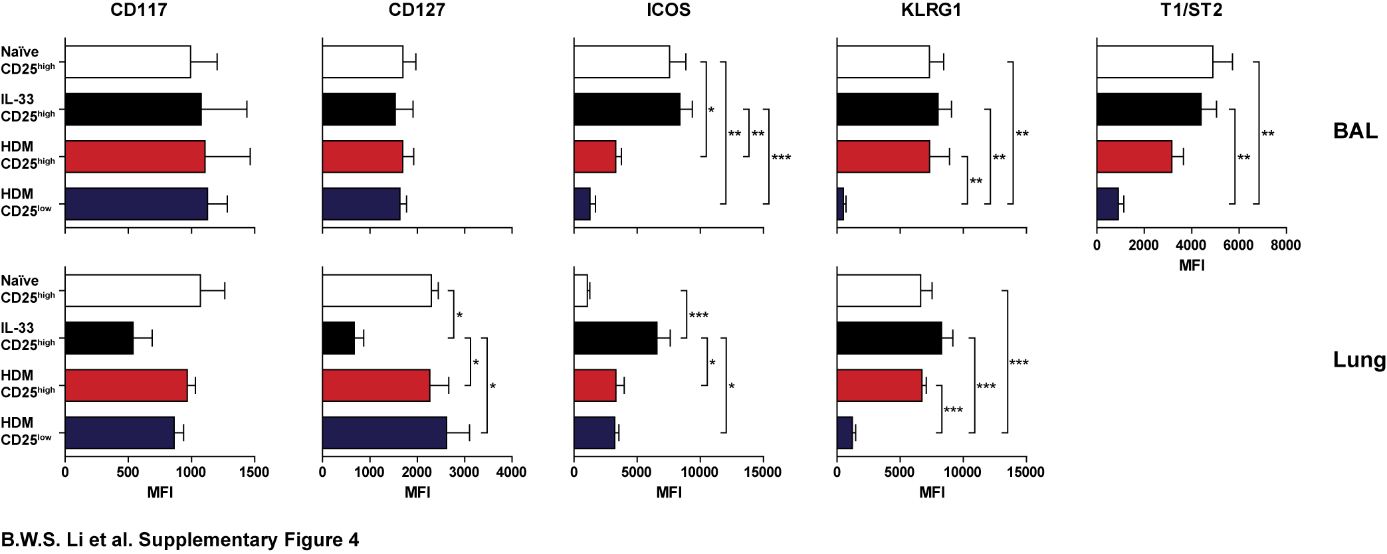
**

**Figure S4. Characterization of phenotypic heterogeneity of ILC2s in BAL and lung in airway inflammation.**

Analysis of the MFI values of CD117, IL-7R (CD127), ICOS, KLRG1 and IL-33R (T1/ST2), comparing naïve ILC2s (*white bars*) and IL-33-activated ILC2s (*black bars*) with CD25^high^ (*red bars*) and CD25^low^ (*blue bars*) ILC2s in HDM-driven airway inflammation in BAL fluid and lung from GATIR mice. T1/ST2 expression on lung ILC2s was very low in all conditions. Data are shown as mean + SEM of n = 4-6 mice per group of a single experiment and are representative of two independent experiments. * p ≤ 0.05, ** p ≤ 0.01, *** p ≤ 0.001.

**
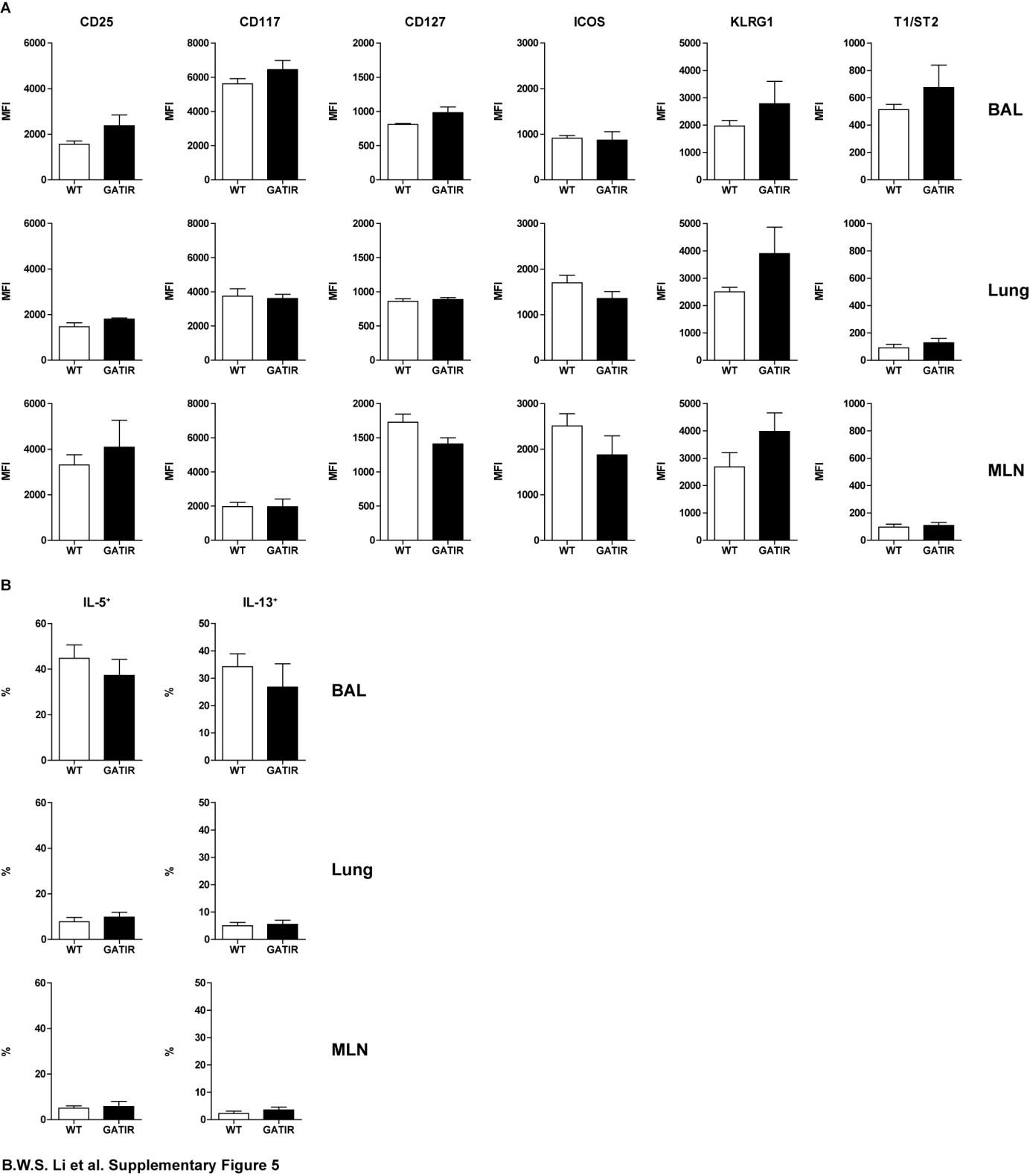
**

**Figure S5. Comparison of surface marker expression profile on ILC2s from WT and GATIR mice.**

Quantification of (A) MFI values of ILC2 surface markers and (B) IL-5 and IL-13 production by ILC2s in BAL fluid, lung and mediastinal lymph node (MLN) of HDM-treated GATIR mice and WT controls. Data are shown as mean + SEM of n = 4 mice per group of a single experiment and are representative of two independent experiments.

**
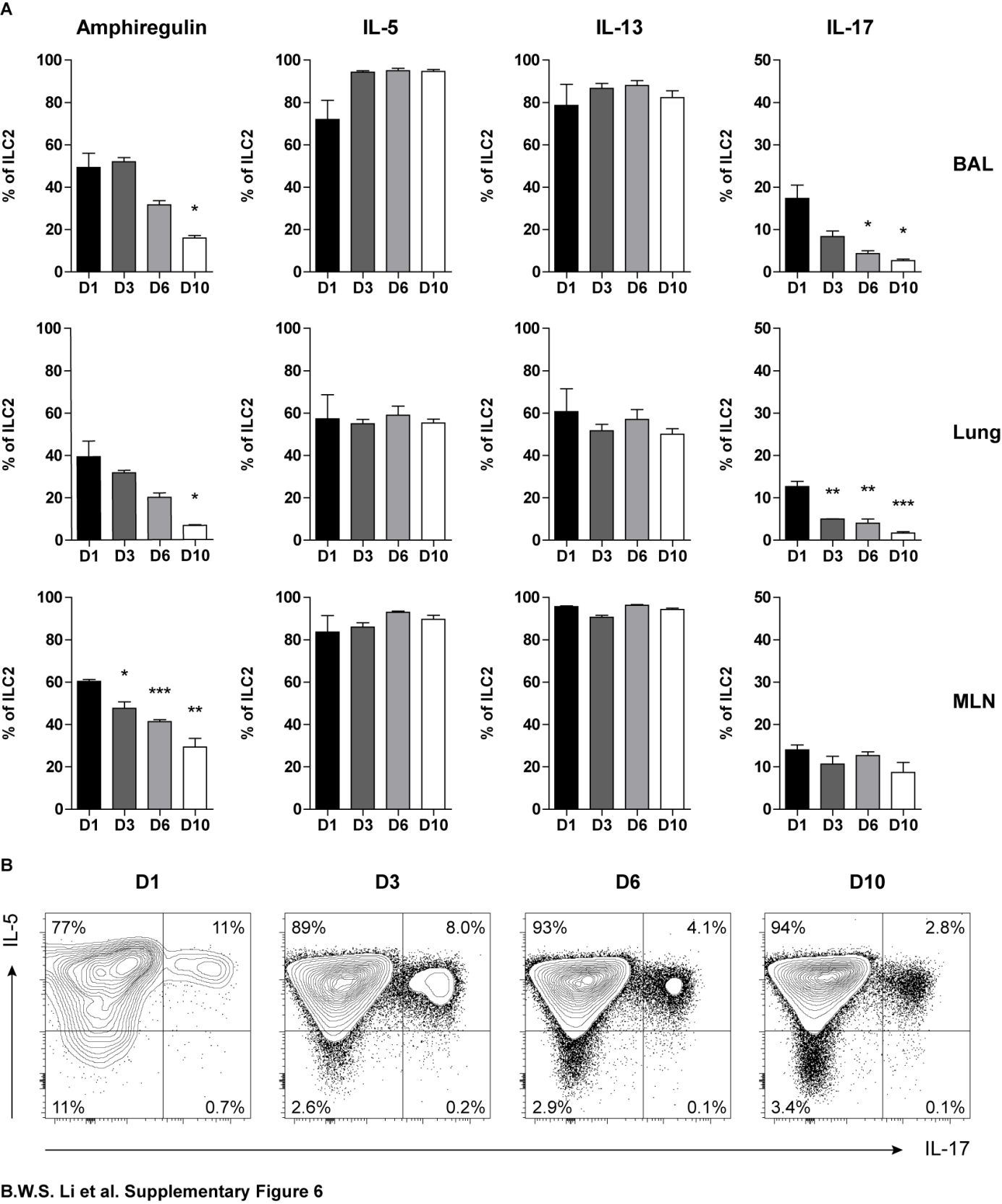
**

**Figure S6. IL-33-activated ILC2s co-express IL-5, IL-13 and IL-17.**

(A) Quantification of ILC2 cytokine production over time in BAL fluid, lung and MLN of IL-33-treated GATIR mice. Data are shown as mean + SEM of n = 3 mice per group of a single experiment. * p ≤ 0.05, ** p ≤ 0.01, *** p ≤ 0.001 compared to D1 unless otherwise indicated.

(B) Flow cytometric analysis of IL-5 and IL-17 production over time in BAL fluid ILC2s of IL-33-treated GATIR mice. Plots represent combined data using the concatenate option in FlowJo (n = 3).

**
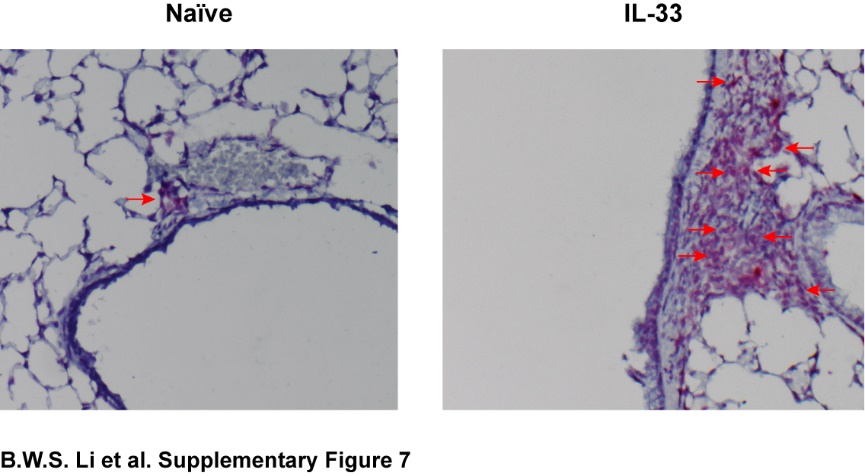
**

**Figure S7. Accumulation of *Gata3*-expressing cells after IL-33 stimulation.**

*In situ* hybridization assay of *Gata3* mRNA on lungs from naïve and IL-33-treated WT mice. Red arrows indicate *Gata3* mRNA expression. Images are representative of two independent experiments.

Table S1 Antibodies used for flow cytometry.

| **Antibody** | **Conjugate** | **Clone** | **Company** |
| --- | --- | --- | --- |
| Amphiregulin | Biotin | Polyclonal | R&D Systems |
| B220 | PE | RA3-6B2 | eBioscience |
| CD117 | Brilliant Violet 650 | 2B8 | BD Biosciences |
| CD11b | PE | M1/70 | eBioscience |
| CD11b | PerCP-Cy5.5 | M1/70 | BD Biosciences |
| CD11b | Alexa Fluor 700 | M1/70 | eBioscience |
| CD11c | PE | N418 | eBioscience |
| CD11c | PE-Texas Red | N418 | Invitrogen |
| CD11c | eFluor 450 | N418 | eBioscience |
| CD127 | PE-Cy7 | A7R34 | eBioscience |
| CD127 | eFluor 450 | A7R34 | eBioscience |
| CD19 | PE | 1D3 | BD Biosciences |
| CD19 | PerCP-Cy5.5 | eBio1D3 | eBioscience |
| CD19 | Biotin | 1D3 | BD Biosciences |
| CD19 | Alexa Fluor 700 | eBio1D3 | eBioscience |
| CD25 | PerCP-Cy5.5 | PC61.5 | eBioscience |
| CD25 | Brilliant Violet 605 | PC61 | BioLegend |
| CD3 | PE | 145-2c11 | eBioscience |
| CD3 | PE-CF594 | 145-2c11 | BD Biosciences |
| CD4 | Alexa Fluor 700 | GK1.5 | eBioscience |
| CD4 | PerCP-Cy5.5 | RM4-5 | eBioscience |
| CD4 | Brilliant Violet 605 | RM4-5 | BD Biosciences |
| CD4 | Brilliant Violet 711 | RM4-5 | BD Biosciences |
| CD45 | PE-CF594 | I3/2.3 | Abcam |
| CD45 | Pe-Cy7 | 30-F11 | eBioscience |
| CD5 | PE | 53-7.3 | eBioscience |
| CD8 | APC | 53-7.3 | eBioscience |
| CD8 | APC-EF780 | 53-7.3 | eBioscience |
| CD8 | PE | 53-6.7 | eBioscience |
| CD86 | PE-Cy7 | GL1 | BD Biosciences |
| CD90.2 | FITC | 53-2.1 | BD Biosciences |
| FcεRIα | PE | MAR-1 | eBioscience |
| FoxP3 | Alexa Fluor 700 | FJK-16s | eBioscience |
| FoxP3 | PE-Cy7 | FJK-16s | eBioscience |
| Gata3 | eFluor 660 | TWAJ-14 | eBioscience |
| Gr-1 | PE | RB6-8C5 | BD Biosciences |
| Gr-1 | APC-eFluor 780 | RB6-8C5 | eBioscience |
| ICOS | APC | C398.4A | eBioscience |
| ICOS | PE-Cy7 | 7E.17G9 | eBioscience |
| IFN-γ | APC | XMG1.2 | BD Biosciences |
| IFN-γ | Brilliant Violet 650 | XMG1.2 | BD Biosciences |
| IL-10 | PerCP-Cy5.5 | JES5-16E3 | eBioscience |
| IL-13 | eFluor 450 | eBio13A | eBioscience |
| IL-13 | eFluor 660 | eBio13A | eBioscience |
| IL-17 | Alexa Fluor 700 | TC11-18H10.1 | BD Biosciences |
| IL-4 | Brilliant Violet 711 | 11B11 | BD Biosciences |
| IL-4 | Biotin | BVD4-1D11 | eBioscience |
| IL-4 | PE-Cy7 | 11B11 | BD Biosciences |
| IL-5 | APC | TRFK-5 | BD Biosciences |
| IL-5 | Biotin | TRFK4 | BD Biosciences |
| IL-9 | PerCP-Cy5.5 | D9302C12 | BD Biosciences |
| IL-9 | PE | D9302C12 | BD Biosciences |
| Ki-67 | FITC | SolA15 | eBioscience |
| Ki-67 | Alexa Fluor 700 | SolA15 | eBioscience |
| KLRG1 | APC | 2F1 | BD Biosciences |
| KLRG1 | PE-CF594 | 2F1 | BD Biosciences |
| MHCII | Alexa Fluor 700 | M5/114.15.3 | eBioscience |
| MHCII | Brilliant Violet 650 | M5/114.15.2 | BD Biosciences |
| NK1.1 | PE | PK136 | eBiosciences |
| NK1.1 | APC | PK136 | BD Biosciences |
| RORγt | PE | Q31-378 | BD Biosciences |
| Sca-1 | Pacific Blue | D7 | BioLegend |
| Sca-1 | Brilliant Violet 786 | D7 | BD Biosciences |
| Siglec-F | PE | E50-2440 | BD Biosciences |
| Siglec-F | PE-CF594 | E50-2440 | BD Biosciences |
| Streptavidin | APC-eFluor 780 |  | eBioscience |
| Streptavidin | Brilliant Violet 786 |  | BD Biosciences |
| T1/ST2 | Biotin | DJ8 | MD Bioproducts |
| T1/ST2 | FITC | DJ8 | MD Bioproducts |
| T-bet | Brilliant Violet 421 | O4-46 | BD Biosciences |
| TER-119 | PE | TER-119 | eBioscience |

Table S2 Antibodies used for confocal microscopy.

| **Antibody** | **Conjugate** | **Clone** | **Company** |
| --- | --- | --- | --- |
| B220 | Biotin | TY25 | eBioscience |
| CD3 | Biotin | 145-2C11 | BD Biosciences |
| αrat | Cy5 | Polyclonal | Jackson ImmunoResearch |
| αhamster | Cy3 | Polyclonal | Jackson ImmunoResearch |
